# Curcumin-Loaded Milk-Derived Exosomes Improve the Developmental Competence of Yak Oocytes by Regulating Mitophagy

**DOI:** 10.3390/antiox15080922

**Published:** 2026-07-24

**Authors:** Tingting Lu, Xin Ma, Meng Wang, Yangyang Pan, Xiaoqing Yang, Shantong Qiu, Xueru Yang, Sijiu Yu

**Affiliations:** 1 College of Veterinary Medicine, Gansu Agricultural University, Lanzhou 730070, China; 18693297203@163.com (T.L.); mxinmaxin@163.com (X.M.); wangmeng@gsau.edu.cn (M.W.); 15117062126@163.com (X.Y.); qiushantong1998@163.com (S.Q.); 15352326375@163.com (X.Y.); yusj@gsau.edu.cn (S.Y.); 2 Gansu Province Livestock Embryo Engineering Research Center, Lanzhou 730070, China

**Keywords:** milk-derived exosomes, curcumin, mitophagy, yak, in vitro oocyte maturation, drug delivery

## Abstract

Yaks are a distinctive livestock species native to the Qinghai–Tibet Plateau. However, the low in vitro maturation rate of their oocytes significantly limits the efficiency of assisted reproductive technologies. Curcumin (CUR), known for its bioactive functions, including antioxidant and anti-inflammatory properties, suffers from low water solubility and bioavailability, which restricts its practical applications. This study aimed to develop a curcumin-loaded bovine milk-derived exosome nanodelivery system (CUR-mEXOs) and investigate its effects on the in vitro maturation of yak oocytes and the embryonic development of parthenogenetic embryos, leveraging its natural biocompatibility and targeted delivery properties. The results indicated that the isolated mEXOs exhibited typical exosome morphology and nanoscale particle size characteristics and were effectively internalized by the oocytes. During in vitro maturation, treatment with 10 μM CUR produced optimal outcomes. Compared to free CUR, CUR-mEXOs significantly enhanced the cumulus expansion index and the rate of first polar body expulsion, reduced intracellular ROS accumulation and mitochondrial superoxide levels, and improved mitochondrial function and spindle morphology, while simultaneously upregulating the expression of factors related to mitochondrial autophagy and oocyte maturation. Following intervention with the mitochondrial autophagy inhibitor CsA, the promotive effect of CUR-mEXOs was significantly diminished, leading to increased blastocyst apoptosis and a decrease in the total cell count. In summary, CUR-mEXOs can enhance the quality of in vitro maturation of yak oocytes and their embryonic developmental capacity following parthenogenesis by regulating mitochondrial autophagy. This study established an experimental foundation for optimizing the in vitro maturation system of yak oocytes and developing strategies for the delivery of natural bioactive substances. Additionally, this study provides a theoretical basis for enhancing the efficiency of assisted reproductive technologies in yaks.

## 1. Introduction

Yaks are a unique and valuable livestock species indigenous to the Qinghai–Tibet Plateau. They have long adapted to the extremely high-altitude environment characterized by cold temperatures, low oxygen levels, and intense ultraviolet radiation, endowing them with significant economic value and serving as a vital genetic resource [1]. However, their natural reproductive rate is low, and adverse factors in the high-altitude grazing environment further exacerbate reproductive challenges, hindering population expansion and breeding improvement [2]. With the advancement of assisted reproductive technologies, such as artificial insemination and embryo transfer, the acquisition of high-quality mature oocytes has become crucial for enhancing the efficiency of in vitro embryo production in yaks [3,4].

The developmental potential of oocytes is influenced not only by nuclear maturation but also by the quality of cytoplasmic maturation, with mitochondrial function serving as a key indicator [5,6]. Previous studies have demonstrated that a decrease in mitochondrial membrane potential, excessive accumulation of reactive oxygen species (ROS), or abnormal mitochondrial distribution disrupts the redox homeostasis of oocytes, consequently affecting the maturation quality and embryonic developmental capacity [7,8]. Mitophagy, a form of selective autophagy, plays a crucial role in clearing damaged mitochondria, thereby maintaining mitochondrial quality and energy homeostasis within cells [9]. The PINK1/Parkin pathway is one of the classical regulatory pathways of mitophagy [10]. When mitochondria are damaged or their membrane potential decreases, PINK1 accumulates at the outer mitochondrial membrane and recruits Parkin, which promotes the engulfment of damaged mitochondria by autophagosomes and their subsequent degradation in lysosomes [11,12]. Xu et al. found that resveratrol can alleviate zearalenone-induced mitochondrial damage, oxidative stress, and apoptosis in porcine oocytes by enhancing PINK1/Parkin-mediated mitochondrial autophagy, thereby improving subsequent embryonic development [13]. Other studies have indicated that promoting mitochondrial autophagy in oocytes helps maintain their quality and delays reproductive decline [14]. However, there is currently a lack of systematic research on whether mitochondrial autophagy is sufficiently activated in yak oocytes—particularly under the stressful conditions of in vitro culture—to counteract oxidative stress, and whether this mechanism can serve as a target for enhancing developmental potential.

Curcumin (CUR) is a natural polyphenolic bioactive compound extracted from plants of the Zingiberaceae family, exhibiting a variety of biological activities, including antioxidant, anti-inflammatory, anti-apoptotic, and mitochondrial function-regulating effects [15]. Recent studies have demonstrated that curcumin not only functions as a free radical scavenger but also promotes mitochondrial autophagy by activating the AMPK/PINK1 signaling pathway, thereby effectively alleviating oxidative stress [16]. In the realm of reproductive health, curcumin has been reported to mitigate damage associated with premature ovarian failure and may provide ovarian protection by regulating oxidative stress and apoptosis [17]. Moreover, relevant studies have confirmed that curcumin can reduce oocyte apoptosis by upregulating Bcl-2 expression, alleviating oxidative stress, and maintaining oocyte quality [18]. However, free CUR exhibits poor water solubility, insufficient stability, low bioavailability, and limited cellular delivery efficiency, which restricts its effectiveness in both in vitro culture and in vivo applications [19,20]. Exosomes are cell-secreted nanovesicles that carry various bioactive molecules and participate in intercellular communication [21]. Milk-derived mxosomes (mEXOs) are highly biocompatible, exhibit low immunogenicity, and possess exceptional transmembrane delivery capabilities, making them ideal carriers for drug delivery [22]. Furthermore, their lipid bilayer membrane structure effectively encapsulates hydrophobic active substances, significantly enhancing stability and cellular uptake efficiency [23,24]. Studies have shown that loading curcumin and resveratrol into exosomes protects them from metabolic degradation, thereby facilitating targeted drug delivery [25]. Additionally, utilizing exosomes as carriers can prolong the in vivo half-life of drugs, improving drug stability and bioavailability [26,27]. Therefore, loading curcumin into bovine exosomes is expected to address the issue of insufficient delivery efficiency of free curcumin and enhance its regulatory effects on the oocyte mitochondrial function.

Currently, there is a limited understanding of whether CUR-mEXOs can enhance the in vitro maturation and embryonic developmental potential of yak oocytes by regulating mitochondrial autophagy. Accordingly, this study aims to establish a system utilizing CUR-mEXOs to investigate their effects on the quality of in vitro maturation of yak oocytes. The objective is to elucidate the mechanisms by which CUR-mEXOs improve the developmental potential of yak oocytes, thereby providing new insights into enhancing the quality of in vitro oocyte maturation and optimizing in vitro embryo production systems. Ultimately, this research seeks to advance the development of assisted reproduction and breeding technologies.

## 2. Materials and Methods

### 2.1. Chemicals and Reagents

All chemicals and reagents used in the present study were purchased from Sigma Chemical Co. (St. Louis, MO, USA). Additionally, CUR and the mitochondrial autophagy inhibitor Cyclosporin A (CsA) were acquired from MedChemExpress (MCE) (Monmouth, NJ, USA).

### 2.2. Sample Collection

#### 2.2.1. Milk Sample Collection

Before sampling, the surface of the udder was wiped with warm towels, followed by sequential disinfection using iodine tincture and 75% ethanol. The initial three streams of colostrum were discarded prior to sample collection. Colostrum was collected from healthy yaks under aseptic conditions for the isolation and preparation of milk-derived exosomes. All samples were transferred into sterile containers, refrigerated at 4 °C during transportation to the laboratory, and subjected to subsequent experimental processing.

#### 2.2.2. Oocyte Collection and Processing

Ovarian samples from yaks were collected at a commercial slaughterhouse in Linxia City, Gansu Province. During the estrus period of the yaks, the laboratory received approximately 80 pairs of ovarian samples each day. Following collection, the ovarian samples were placed in a sterile incubator at 32–36 °C and transported to the laboratory within 4 h. Follicles with diameters ranging from 2 to 8 mm were extracted from the surface of the ovaries using sterile 12–18 G needles. Under a stereomicroscope equipped with a heating stage, immature cumulus-oocyte complexes (COCs) with intact morphology and more than three layers of granulosa cells were screened. The COCs were washed three times with a pre-warmed oocyte washing solution (M199 medium + 5% serum) before being transferred to an equilibrated maturation medium (M199 + 10% fetal bovine serum + 100 μg/mL FSH + 50 μg/mL LH + 100 μg/mL penicillin + 100 μg/mL streptomycin). In accordance with the experimental design, each 400 µL of oocyte culture medium contained 50 COCs. CUR, CUR-mEXOs, and the mitochondrial autophagy inhibitor CsA were incorporated into the maturation medium for the respective treatment groups, whereas the control group received an equivalent volume of saline. Culture conditions were maintained at 38.5 °C and 5% CO_2_, and mature cumulus-oocyte complexes were harvested after 24 h. Cumulus cells were removed using 0.1% hyaluronidase, followed by washing with DPBS and gentle aspiration with a pipette to isolate individual oocytes. Oocyte maturity was assessed based on the extrusion of the first polar body, and mature oocytes were collected for parthenogenetic embryo preparation.

### 2.3. Analysis of the Cumulus Expansion Index

After 24 h of in vitro culture, the degree of cumulus expansion was assessed in the COCs. Based on the differences in cumulus cell morphology, cumulus expansion was classified into five stages: Stage 0 indicates no cumulus expansion; Stage 1 denotes expansion limited to the outermost 1–2 layers of cumulus cells; Stage 2 involves radial expansion of the outer cumulus cells; Stage 3 shows full expansion of cumulus cells excluding those within the corona radiata; and Stage 4 exhibits full expansion of all cumulus cells. The Cumulus Expansion Index (CEI) was computed via weighted averaging: the oocyte count of each stage was multiplied by its respective stage score (0–4), and the resulting sum was divided by the total oocyte number within the sample. The calculation formula was shown as follows: CEI = Σ (oocyte number per stage × matching stage score)/total number of oocytes.

### 2.4. Isolation of mEXOs and Preparation of CUR-mEXOs

Milk samples were collected from healthy yaks, and mEXOs were isolated through differential centrifugation. Initially, the milk pH was regulated to 5.0 by the addition of 10% acetic acid (ReagentPlus^®^, ≥99%, A6283, Merck, St. Louis, MO, USA). Subsequently, centrifugation was performed at 4 °C and 16,500× *g* for 30 min to eliminate casein and large cell fragments. The supernatant was then subjected to ultracentrifugation at 4 °C and 110,000× *g* for 2 h. The harvested precipitate was resuspended in PBS and subjected to ultracentrifugation at 4 °C and 110,000× *g* for 1 h. After resuspension in PBS, the supernatant fluid was filtered with 0.22 μm microporous membranes for the isolation of mEXOs. The protein content of mEXOs was determined with a BCA assay kit (P0009, Beyotime, Shanghai, China). Purified exosomal vesicles should be subpackaged before storage and preserved at −80 °C; repeated freeze–thaw cycles must be strictly avoided.

To load mEXOs with CUR, mEXOs were admixed with CUR at a 1:1 mass ratio. Co-incubation method: After thorough mixing, incubate in a shaking incubator at 37 °C for 12 h. Ultrasonication method: After thorough mixing, place the mixture on ice and sonicate at 70 W, cycling 10 s on/10 s off for six cycles. After sonication, the mixed solution was incubated in a shaking incubator at 37 °C for 2 h to stabilize the exosome membranes. For the freeze–thaw treatment, the mixture was snap-frozen in liquid nitrogen (−196 °C) for 3 min, followed by thawing in a 37 °C water bath for 15 min; this freeze–thaw cycle was performed four times. The acquired mixture was spun at 120,000× *g* for 1 h at 4 °C. The pellet was washed with PBS and subjected to high-speed centrifugation under the same conditions to obtain CUR-mEXOs. In this study, the administration concentration of free CUR was set at 10 μM. To match this concentration, CUR-mEXOs were prepared, with a determined protein concentration of 78 μg/mL (the protein concentration of pure milk-derived exosomes, mEXOs, was 626 μg/mL).

### 2.5. Analysis of the Physicochemical Properties of mEXOs and Evaluation of Their Drug-Loading Capacity

Characterization of mEXOs was carried out following the MISEV2018 criteria. Exosomal morphology was visualized by transmission electron microscopy(TEM; H-7500, Hitachi, Tokyo, Japan). Exosome markers were detected using Western blotting, with antibodies against HSP70, TSG101, and CD81, and Calnexin as a negative control. Nanoparticle tracking analysis was utilized to assess particle size distribution and concentration(NTA; ZetaView PMX 110, Particle Metrix, Meerbusch, Germany). The average particle size (nm) and concentration (particles/mL) were quantified with ZetaView software (version 8.05.14 SP7). Exosomal protein content was determined using a BCA assay kit, and CUR (CUR in Exosomes) content was quantified by high-performance liquid chromatography (HPLC; Ultimate 3000, Thermo, Waltham, MA, USA) using a Welch Ultimate PLUS C18 column (250 × 4.6 mm, 5 μm). The detection conditions were as follows: wavelength 280 nm, flow rate 1.0 mL/min, column temperature 35 °C, and injection volume 5 μL. CUR standard solutions (4, 8, 16, 32, 64 mg/L) were utilized to establish the calibration curve. This was performed to evaluate the loading efficiency of different drug-loading methods (co-incubation, repeated freeze–thaw, and sonication). The loading capacity (LC) and encapsulation efficiency (EE) were calculated using the following formulas: LC (%) = (the mass of encapsulated CUR/the total protein content of CUR-mEXOs) × 100% and EE (%) = (the mass of encapsulated CUR/the mass of CUR initially added) × 100%.

### 2.6. Cell Uptake Assay

The PKH26 fluorescent labeling method was employed to evaluate the cellular uptake of mEXOs and CUR-mEXOs by the yak oocytes. A staining working solution was prepared following the instructions provided in the PKH26 staining kit (MINI26, Sigma-Aldrich, St. Louis, MO, USA). mEXOs and CUR-mEXOs were incubated with the PKH26 working solution for 10 min at 37 °C in the dark. To eliminate unbound dye, the labeled exosomes were resuspended in PBS, spun at 120,000× *g* for 1 h at 4 °C, and the acquired pellet underwent three rounds of PBS washing. Subsequently, yak oocytes and labeled exosomes were co-incubated at 38.5 °C and 5% CO_2_ atmosphere for 24 h, after which they were fixed with 4% paraformaldehyde for 30 min. Fluorescence images were captured using a holographic tomographic cell imaging system (HT-X1, Baitai Technology, Shanghai, China).

### 2.7. Parthenogenetic Activation and Embryo Culture

**Figure 1:**
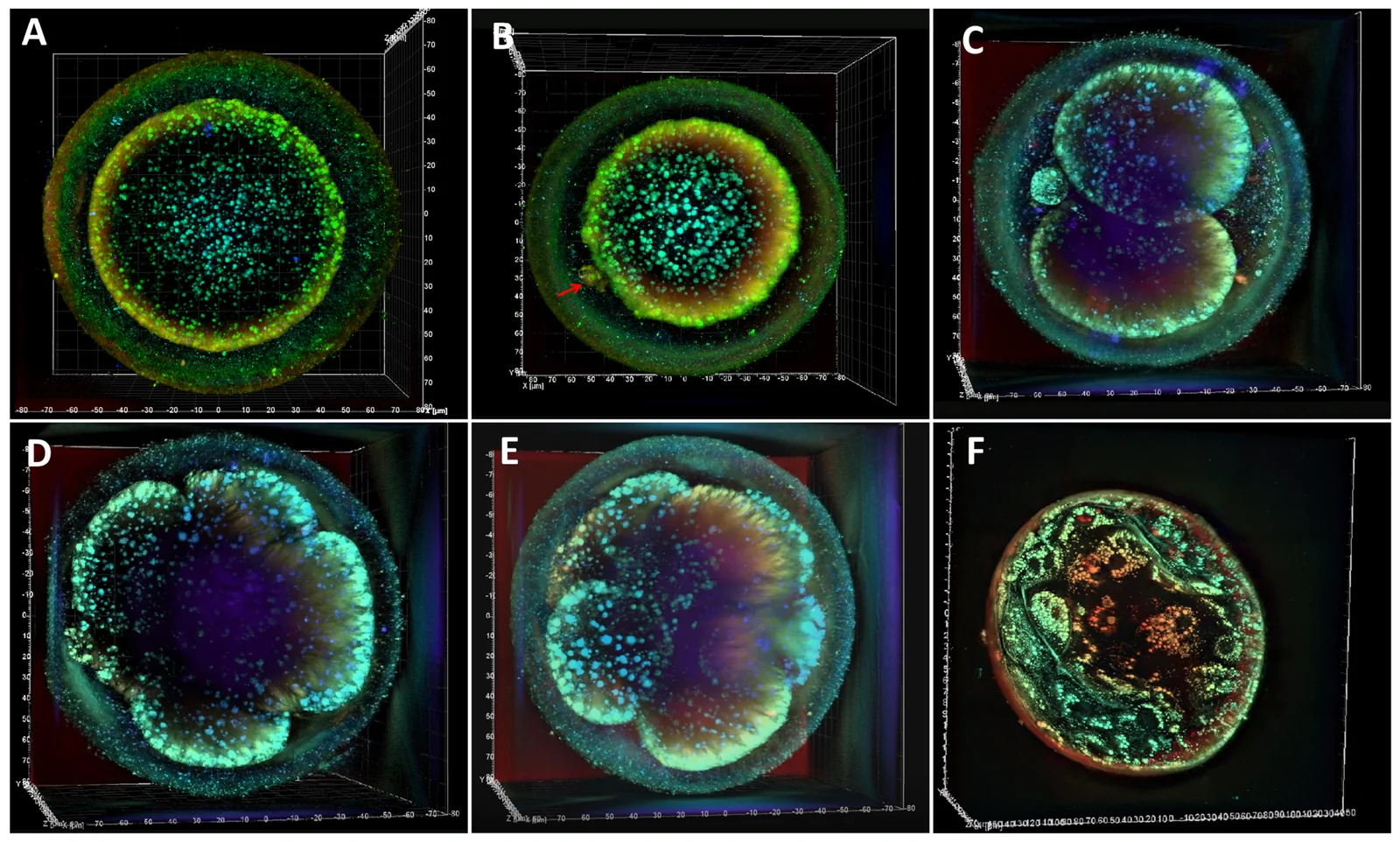
Maturation stages of yak oocytes and representative pseudocolored 3D confocal images of embryonic development following parthenogenetic activation. (**A**) Immature oocyte; (**B**) Mature oocyte releasing the first polar body (indicated by the red arrow); (**C**–**F**) Show the developmental stages of parthenogenetic embryos at the 2-cell stage (**C**), 4-cell stage (**D**), 8-cell stage (**E**), and blastocyst stage (**F**), respectively.

Oocytes that underwent in vitro maturation were collected and randomly assigned to different treatment groups, with three biological replicates per group. Each replicate sample contained 300 oocytes. After washing the oocytes three times with DPBS, 50 oocytes were kept at −80 °C for later real-time quantitative PCR assays, another 50 oocytes were washed with DPBS and transferred to immunofluorescence fixative for staining, and the remaining 100 oocytes were used to evaluate the developmental competence of yak oocytes. After three washes with DPBS, the oocytes were transferred to a modified synthetic oviduct fluid (mSOF) medium. Subsequently, under light-protected conditions, the oocytes were treated with 5 μM ionomycin for 5 min and then immediately placed in mSOF medium containing 2 mM 6-dimethylaminopurine (6-DMAP) for culture at 38.5 °C for 4–6 h. Activated embryos were washed three times with pre-warmed DPBS, with each wash lasting for 5 min. The embryos were then transferred to 35 mm embryo culture dishes for further culturing. Three equilibrated droplets of G1 culture medium were prepared in advance in each dish, and 15 embryos were placed in each drop and overlaid with mineral oil. The embryos were incubated at 38.5 °C under saturated humidity and 5% CO_2_. Yak embryo development was observed and recorded at 48, 72, 96, and 168 h post-culture (Figure 1).

### 2.8. Reverse Transcription Quantitative Real-Time Polymerase Chain Reaction (RT-qPCR)

Total RNA was isolated from 50 oocytes per group using the Micro Eazy Total RNA Kit (Omega, Norcross, GA, USA). cDNA was generated through reverse transcription with the GoScript Reverse Transcription Kit (Promega, Madison, WI, USA). The coding sequences (CDS) of the target genes were selected for RT-qPCR analysis based on yak mRNA sequences available in GenBank. Primer Premier 6.0 was utilized to design all primers, which were manufactured by Sangon Biotech(Shanghai, China). (Table 1 for specific sequences). The real-time PCR reaction mixture comprised 1.5 µL cDNA, 0.8 µL of both forward and reverse primers, and 10 µL of SYBR Green II Quantitative PCR Mix (2×), diluted to a final volume of 20 µL using deionized water. Real-time quantitative PCR was conducted using a Roche 480 instrument (Basel, Switzerland) under the following cycling conditions: 30 s of pre-denaturation at 95 °C, followed by 5 s of denaturation at 95 °C, 34 s of annealing at 60 °C, and 30 s of extension at 72 °C, for a total of 45 cycles. *β-actin* was selected as the reference gene, and the saline group was set as the control group. The mRNA transcript levels of all treatment groups were assessed via relative quantification according to the 2^−ΔΔCt^ formula.

### 2.9. Immunofluorescence Staining

Oocytes and embryos were collected from each group, with a total of five oocytes per group, and immobilized with 4% paraformaldehyde at room temperature for 1 h. Following fixation, permeabilization was performed using 0.5% Triton X-100 for 30 min. To prevent nonspecific antibody binding, the samples were incubated in 8% BSA blocking solution for 1 h. After blocking, the samples were immersed in a primary antibody dilution solution and incubated overnight at 4 °C. The next day, the samples were treated with matching fluorescent secondary antibodies and kept in darkness at room temperature for 1 h. Three full DPBS washes were performed prior to adding 2.5 ng/mL DAPI stain. After 3–5 min of light-shielded incubation at room temperature, specimens were rinsed three times again. Finally, fluorescence images were acquired using the GE DeltaVision Elite live-cell imaging system.

### 2.10. Western Blot Analysis

Precipitates of mEXOs and CUR-mEXOs were resuspended in pre-chilled RIPA lysis buffer (R0010, Solarbio, Beijing, China) to extract total protein. Total protein content was quantified with the BCA protein quantification kit. Equal volumes of protein were denatured at 100 °C for 10 min, followed by SDS-PAGE separation and transfer to a PVDF membrane. The membrane was blocked with 5% skim milk at room temperature for 2 h to reduce nonspecific binding and then washed three times with PBST. Subsequently, the membrane was incubated at 4 °C overnight with exosome-positive marker proteins HSP70 (1:1000, bs-0244R, Bioss, Beijing, China), TSG101 (1:1000, bs-52746R, Bioss, Beijing, China), CD81 (1:1000, bs-6934R, Bioss, Beijing, China), and the negative control protein Calnexin (1:5000, 10427-2-AP, Proteintech, Chicago, IL, USA). After incubation, the samples were washed thrice with PBST, then HRP-tagged secondary antibody (1:8000, 8889S, Cell Signaling Technology, Danvers, MA, USA) was supplemented for 2 h incubation at ambient temperature. Protein signals were detected via a chemiluminescence detector.

### 2.11. Detection of Reactive Oxygen Species (ROS) Levels in Oocytes

Following three rounds of rinsing the oocytes in 0.5% PVP-PBS, the specimens were transferred into the DCFH-DA working solution (1:5000, S80033, Beyotime, Shanghai, China). The specimens were then cultured in a CO_2_ incubator for 20 min, after which fluorescent images were captured with an inverted fluorescence microscope.

### 2.12. Mitochondrial Distribution Analysis in Oocytes

MitoTracker, a mitochondria-specific fluorescent probe provided by Invitrogen (Carlsbad, CA, USA), was utilized to observe the spatial arrangement of mitochondria within the oocytes. A total of 50 µg of lyophilized probe powder was dissolved in DMSO to create a 1 mM stock solution. This stock solution was subsequently diluted at a ratio of 1:5000 before being added to the oocyte specimens, followed by a 20 min incubation period. After staining, fluorescent images were captured using an inverted fluorescence microscope.

### 2.13. Oocyte Mitochondrial ROS Quantification

All oocyte specimens underwent three rounds of rinsing with 0.5% PVP-PBS buffer before being transferred to HBSS medium containing 5 µM MitoSOX Red (HYD1055, MCE, Monmouth, NJ, USA). The specimens were maintained under light-shielded conditions at 37 °C for a 20 min staining period. After staining, excess unbound fluorescent reagent was removed by rinsing the oocytes three times. Subsequent fluorescence images of the specimens were captured using an inverted fluorescence microscope.

### 2.14. Evaluation of Apoptosis Levels

The TUNEL staining kit (11684795910, Roche, Basel, Switzerland) was utilized to evaluate apoptosis levels in samples from each treatment group. Specimens were first soaked in 4% paraformaldehyde for 1 h to stabilize cellular structures, then treated with 0.5% Triton X-100 to achieve membrane permeabilization within 30 min. In accordance with the kit instructions, 10 μL of enzyme solution was thoroughly mixed with 90 μL of labeling solution to prepare a working solution. The specimens were subjected to triple rinses with 0.5% PVP-PBS buffer prior to their transfer into the TUNEL working reagent, followed by a 20 min incubation in a CO_2_ incubator. Once the reaction was complete, DAPI dye was utilized to label the cell nuclei, and the samples were maintained under light-shielded ambient conditions for 5 min. Subsequently, three additional rounds of rinsing with 0.5% PVP-PBS were performed, and fluorescent images of the specimens were captured using an inverted fluorescence microscope.

### 2.15. Statistical Analysis

Each experimental treatment was conducted with a minimum of three biological replicates. Statistical analyses of all data were performed using SPSS 25.0 software (Statistical Products and Services, Inc., Cary, IL, USA). The Student’s *t*-test was employed to compare indicators between the two groups. One-way analysis of variance (ANOVA) was used to assess differences among multiple groups, followed by Tukey’s test for subsequent pairwise comparisons. The threshold for statistical significance was set at *p* < 0.05. All measured data are presented as the mean ± standard error of the mean (mean ± SEM).

## 3. Results

### 3.1. Preparation, Characterization, and Uptake of CUR-mEXOs

**Figure 2:**
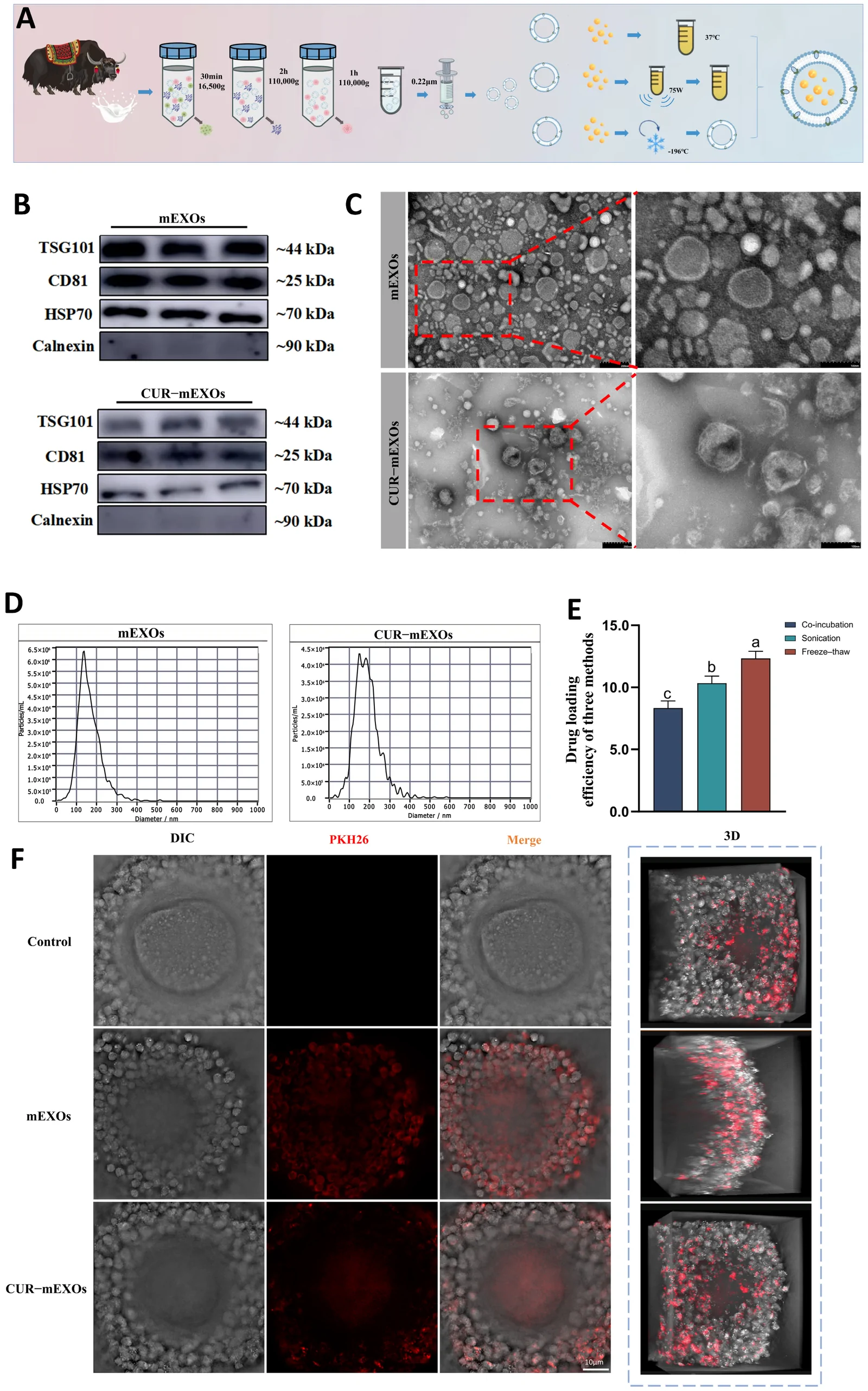
Analysis of physicochemical properties, drug-loading efficiency, and cellular uptake of mEXOs and CUR-mEXOs. (**A**) Exosome isolation protocol; (**B**) Western blot; (**C**) Transmission electron microscopy; (**D**) Nanoparticle tracking analysis; (**E**) Comparison of drug loading efficiency among different loading methods; (**F**) Uto COCs uptake of exosomes. Scale bar = 10 µm. Bars labeled with distinct lowercase letters denote significant statistical discrepancies (*p* < 0.05).

In this study, mEXOs were successfully isolated using differential centrifugation following the addition of 10% acetic acid (Figure 2A). Exosomes with a protein concentration of approximately 6000 μg/mL were isolated from roughly 8 L of milk, then aliquoted and stored at −80 °C; repeated freeze–thaw cycles should be strictly avoided. TEM revealed that both mEXOs and CUR-mEXOs exhibited characteristic cup-shaped double-membrane vesicle structures (Figure 2C). NTA demonstrated that mEXOs had an average particle size of 135.6 nm, whereas CUR-mEXOs had an average particle size of 151.6 nm. Both the particle concentration and size distribution were consistent with the characteristics of the exosomes (Figure 2D). Western blot analysis indicated that the samples exhibited high expression levels of the exosome marker proteins HSP70, CD81, and TSG101, while the endoplasmic reticulum marker protein Calnexin was absent (Figure 2B). This study compared the drug-loading efficiency of three methods: co-incubation, sonication, and repeated freeze–thaw for curcumin-loaded bovine mEXOs. The results (Figure 2E) indicated that the repeated freeze–thaw method yielded a higher drug-loading efficiency than sonication and co-incubation; HPLC analysis showed CUR-mEXOs prepared via repeated freeze–thaw cycles possessed the maximum LC and EE of 13.02% and 7.8% respectively; thus, the repeated freeze–thaw method was selected for subsequent experiments. Furthermore, under a holographic 3D confocal microscope, it was observed that after COCs with PKH26-labeled mEXOs and CUR-mEXOs for 24 h, distinct fluorescent signals were detected within the oocyte cytoplasm, demonstrating that both types of exosomes were efficiently taken up by the cells (Figure 2F). In summary, CUR loading did not alter the fundamental structure or characteristics of the exosomes.

### 3.2. Effects of Various CUR Concentrations on Yak Oocytes

**Figure 3:**
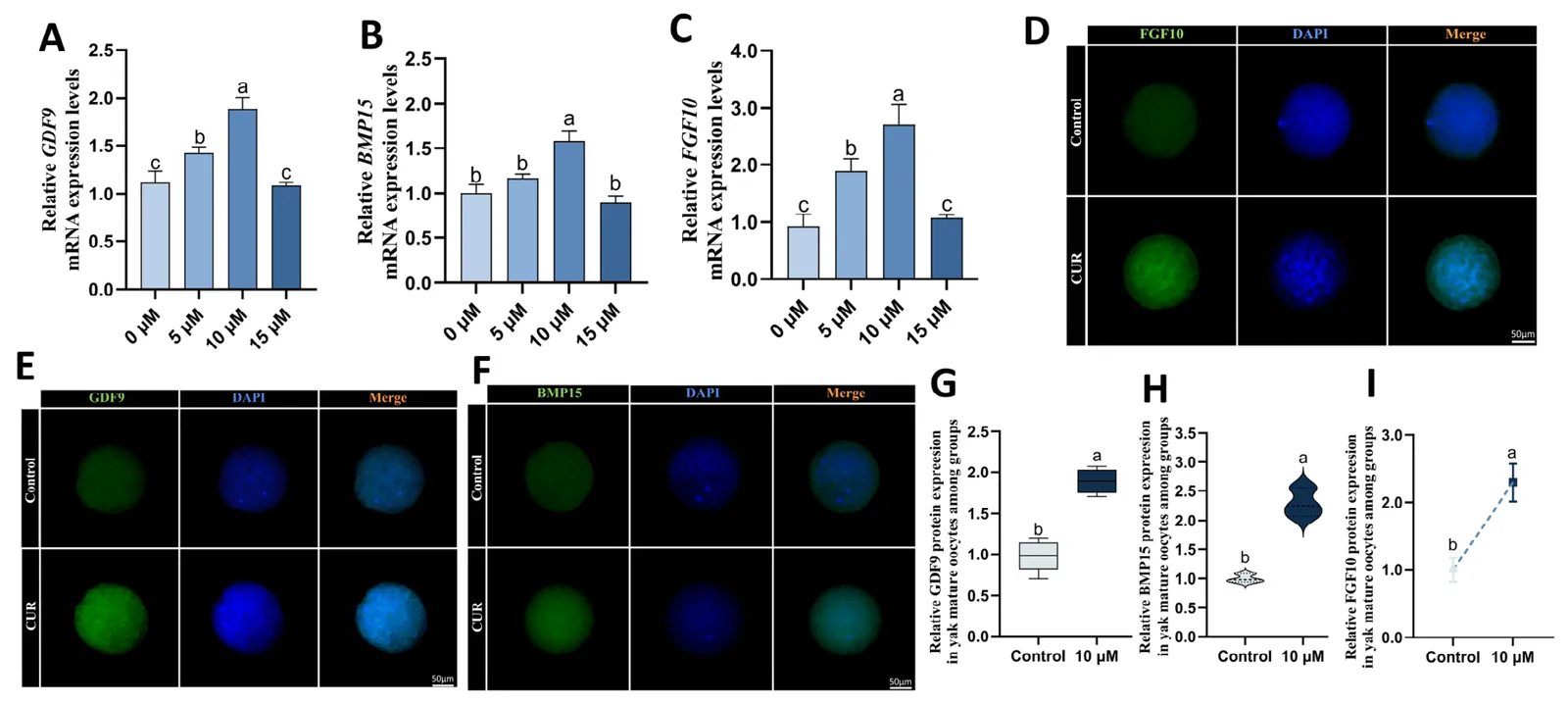
Impacts of various CUR concentrations on oocyte-secreted factors. (**A**–**C**) mRNA expression levels of *GDF9*, *BMP15*, and *FGF10* in mature yak oocytes from different treatment groups; (**D**–**F**) immunofluorescence staining results for proteins in mature yak oocytes from different treatment groups; (**G**–**I**) Relative protein expression levels in different treatment groups of mature yak oocytes. Scale bar = 50 µm. Lines in small trapezoidal plots represent medians. Bars labeled with distinct lowercase letters denote significant statistical discrepancies (*p* < 0.05).

**Figure 4:**
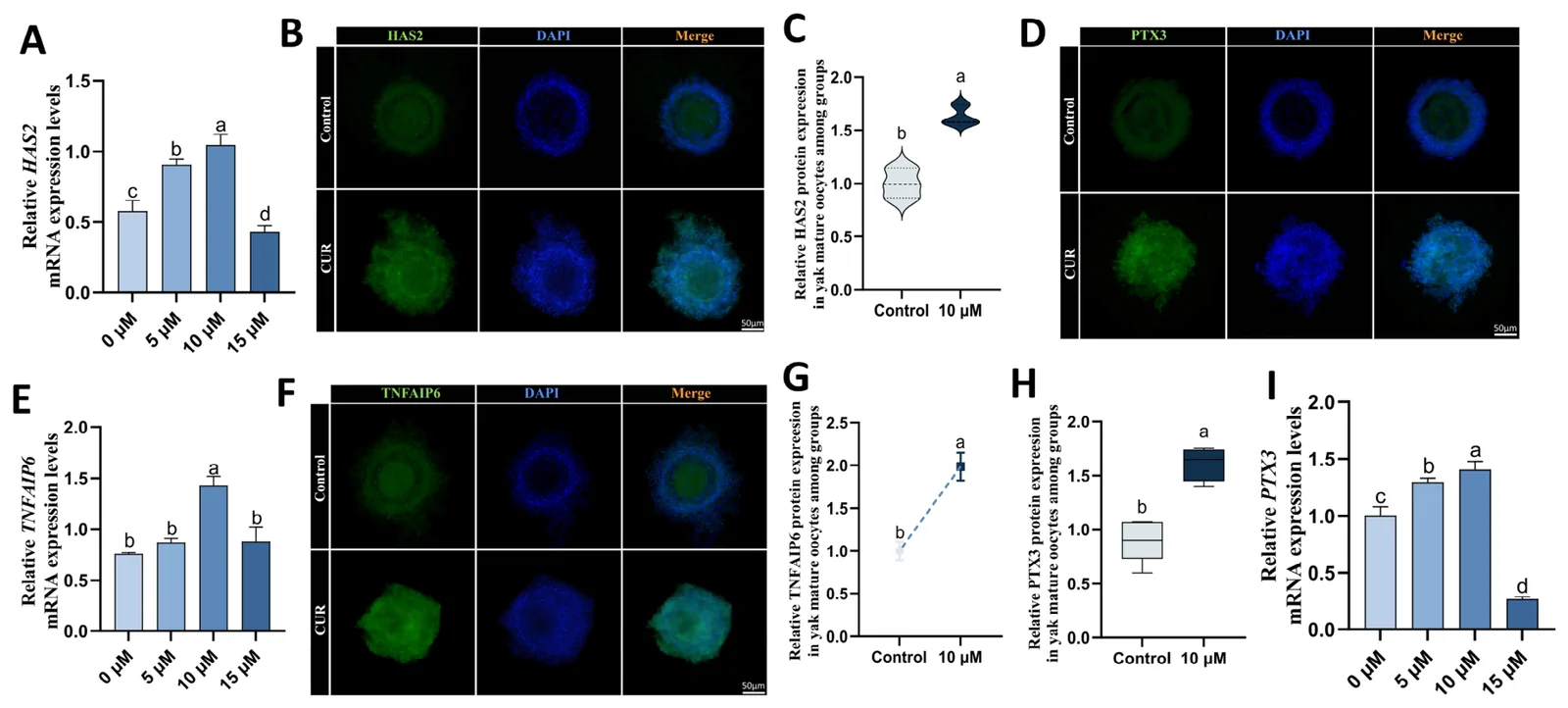
Effects of different concentrations of CUR on cumulus expansion factors. (**A**,**E**,**I**) Quantitative detection of *HAS2*, *PTX3* and *TNFAIP6* mRNA in mature yak oocytes subjected to various treatments; (**B**,**D**,**F**) immunofluorescence staining results of proteins in different treatment groups of mature yak oocytes; (**C**,**G**,**H**) Relative protein expression levels in different treatment groups of mature yak oocytes. Scale bar = 50 µm. Lines in small trapezoidal plots represent medians. Bars labeled with distinct lowercase letters denote significant statistical discrepancies (*p* < 0.05).

We observed the expulsion of the first polar body and the expansion of the cumulus following oocyte maturation, while also detecting factors associated with this process. CUR at varying concentrations (0 μM, 5 μM, 10 μM, and 15 μM) was incorporated into the in vitro maturation medium for yak oocytes. Oocyte maturation was subsequently observed and quantified after 24 h of culture. RT-qPCR results indicated that the mRNA expression of oocyte-secreted factors Growth differentiation factor-9 (*GDF9*), Bone morphogenetic protein 15 (*BMP15*), and Fibroblast growth factor 10 (*FGF10*), along with cumulus expansion factors Hyaluronan synthase 2 (*HAS2*), pentraxin 3 (*PTX3*), and tumor necrosis factor alpha-induced protein 6 (*TNFAIP6*) was significantly elevated in the 10 μM CUR-treated yak oocyte group compared to the control group (*p* < 0.05) (Figure 3 and Figure 4). In contrast, no significant differences in oocyte maturation rates were noted in the 5 μM and 15 μM CUR-treated groups. Consequently, the 10 μM concentration was selected for immunofluorescent labeling of proteins in mature yak oocytes (Figure 3 and Figure 4). The data illustrated that 10 μM CUR significantly upregulated protein levels (*p* < 0.05), and all subsequent experiments utilized a 10 μM concentration of CUR.

### 3.3. Comparison of the Effects of CUR and CUR-mEXOs on Yak Oocytes

**Figure 5:**
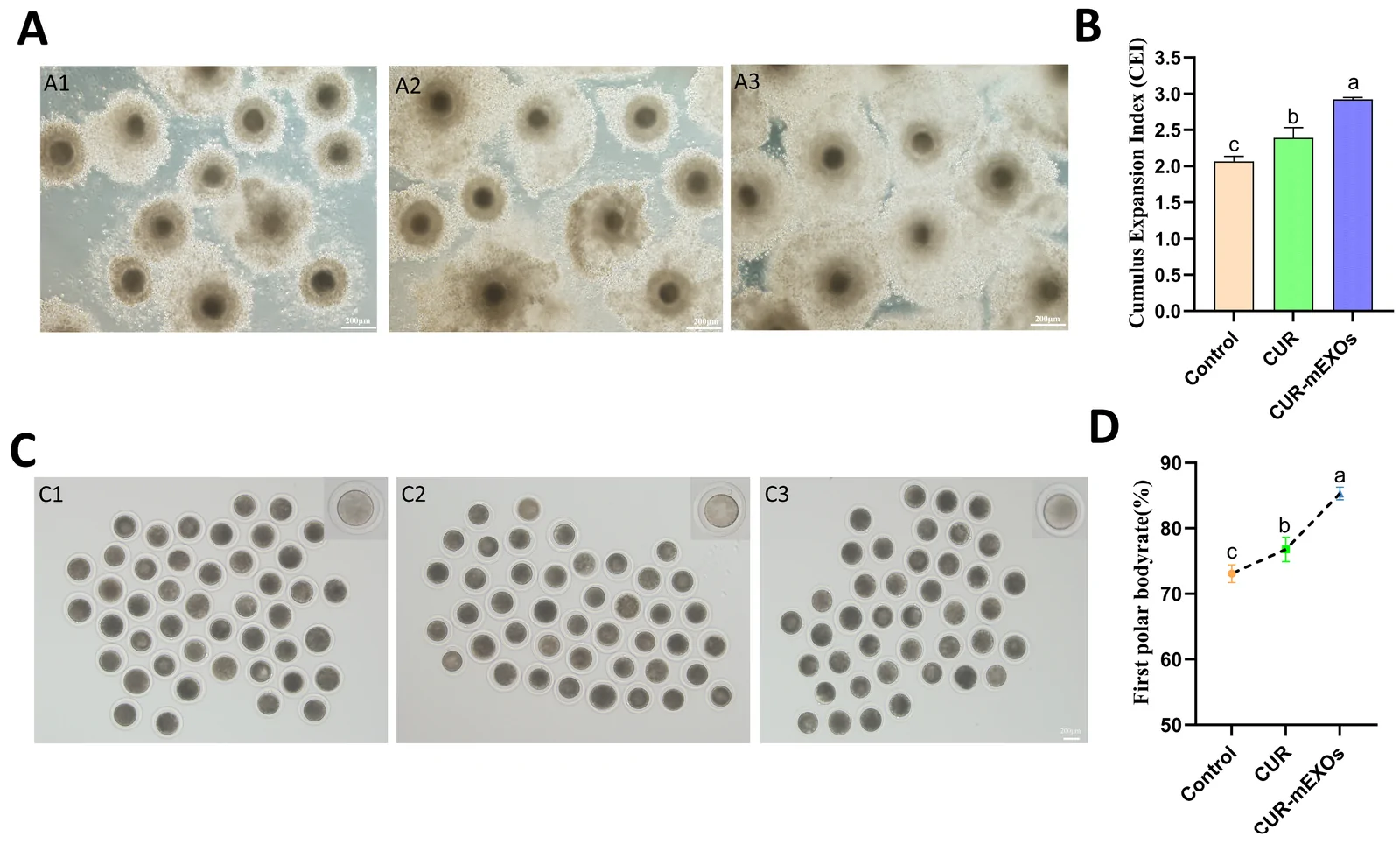
Impacts of CUR-mEXOs on cumulus expansion index and first polar body extrusion ratio of yak oocytes. (**A**) Cumulus expansion morphology of oocyte samples under various treatments. (**A1**) Mature COCs from the blank control group; (**A2**) Mature COCs subjected to CUR treatment; (**A3**) Mature COCs cultured with CUR-mEXOs; (**B**) Statistical quantification of cumulus expansion index for mature yak oocytes across all treatment groups; (**C**) Representative images displaying oocytes with extruded first polar bodies in each group: (**C1**) Oocytes from the control group; (**C2**) CUR group; (**C3**) CUR-mEXOs group; (**D**) The statistical ratio of first polar body extrusion in mature yak oocytes of every group. Scale bar = 200 µm. Bars labeled with distinct lowercase letters denote significant statistical discrepancies (*p* < 0.05).

To evaluate the effects of CUR and CUR-mEXOs treatments on the quality of in vitro maturation of yak oocytes, this study examined the cumulus expansion of oocytes in different treatment groups 24 h post-initiation of in vitro maturation and calculated the cumulus expansion index (Figure 5A). Data revealed that relative to the blank control group, CUR treatment significantly increased the cumulus expansion index, while the CUR-mEXOs group exhibited an even greater increase, surpassing that of the free CUR group (*p* < 0.05) (Figure 5B). This finding suggests that CUR-mEXOs are more effective in promoting yak cumulus-oocyte complex expansion. Furthermore, cumulus cells were enzymatically removed using hyaluronidase to obtain naked oocytes, and the expulsion of the first polar body was observed under a stereomicroscope (Figure 5C). It was observed that CUR supplementation markedly raised the first polar body extrusion rate compared with untreated specimens, whereas the CUR-mEXOs group demonstrated the highest extrusion rate (*p* < 0.05) (Figure 5D).

### 3.4. The Effects of CUR and CUR-mEXOs on the Developmental Potential of Yak Oocytes

**Figure 6:**
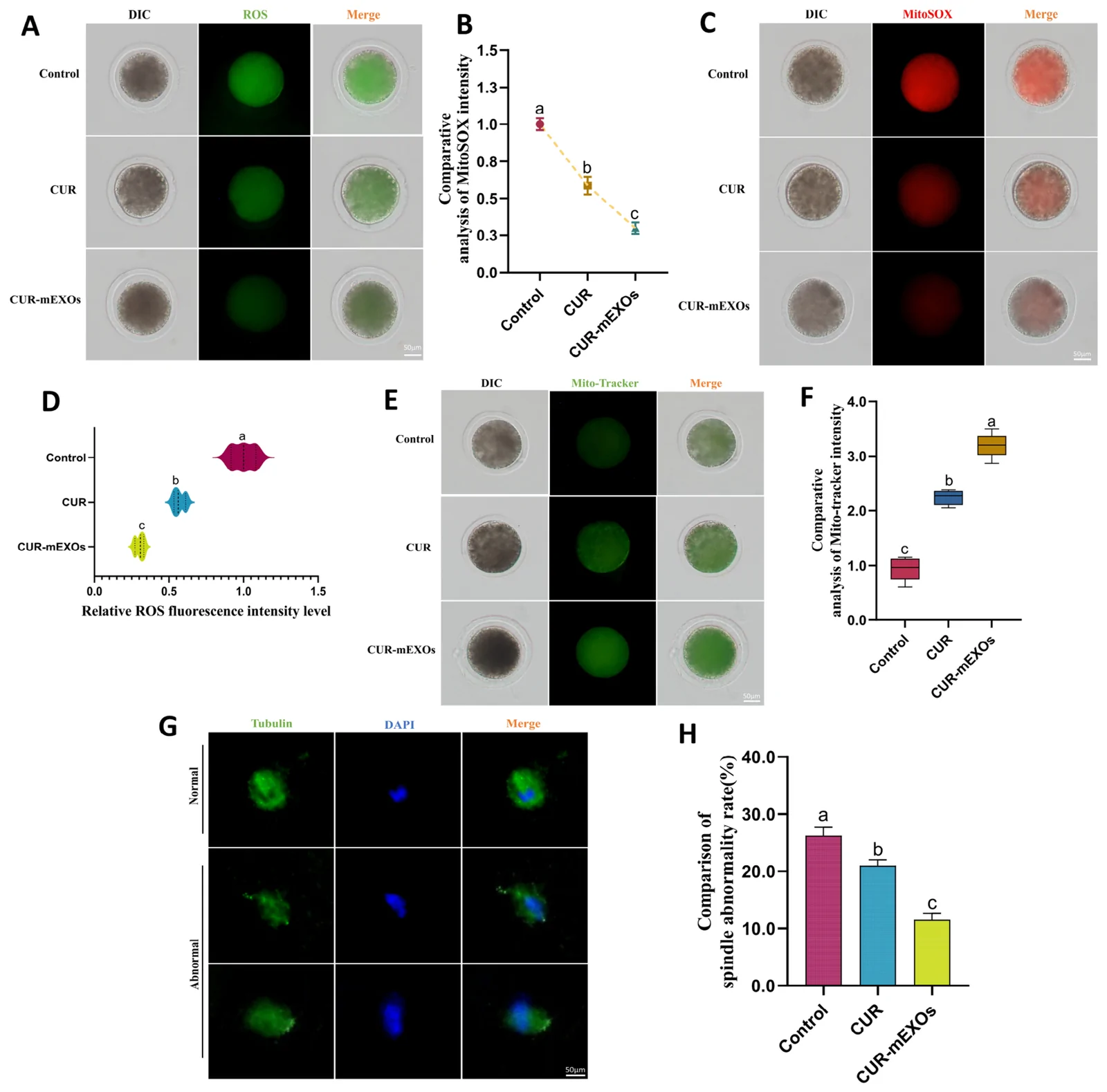
Effects of CUR and CUR-mEXOs on the developmental competence of yak oocytes. (**A**) Changes in intramitochondrial reactive oxygen species (ROS) abundance within oocytes under varied treatments; (**B**) Quantified MitoSOX Red fluorescent signal intensity of oocyte specimens from each group; (**C**) Mitochondrial probe staining outcomes of oocytes subjected to distinct interventions; (**D**) Relative ROS fluorescence readings for oocytes in all treatment groups; (**E**) Variations in the spatial arrangement of mitochondria inside oocytes among different treatments; (**F**) Statistical quantification of mitochondrial fluorescence intensity across all oocyte groups; (**G**) Representative images of spindle morphology in yak oocytes; (**H**) Quantitative evaluation of abnormal spindle proportions in oocytes from each experimental group. Scale bar = 50 µm. Lines in small trapezoidal plots represent medians. Bars labeled with distinct lowercase letters denote significant statistical discrepancies (*p* < 0.05).

The levels of reactive oxygen species (ROS) in oocytes from each experimental group were measured using the DCFH staining method to evaluate the extent of cellular oxidative stress (Figure 6A). The findings indicated that CUR treatment significantly decreased ROS levels in oocytes, with the inhibitory effect of CUR-mEXOs being even more pronounced (*p* < 0.05, Figure 6D). Mitochondria are essential for the developmental potential of oocytes. MitoTracker-specific probes were utilized to assess mitochondrial distribution in oocytes (Figure 6E). In the control group, mitochondrial fluorescence signals were weak and unevenly distributed; conversely, the CUR group exhibited increased mitochondrial fluorescence intensity compared to the control group. The CUR-mEXOs group displayed the strongest fluorescence signal, characterized by a more uniform mitochondrial distribution and significantly higher fluorescence intensity than the control group (*p* < 0.05, Figure 6F). JC-1 staining demonstrated that CUR-mEXOs significantly restored the mitochondrial membrane potential by increasing the red/green fluorescence ratio(Figure S1). Additionally, MitoSOX staining was employed to measure the superoxide levels originating from the oocyte mitochondria (Figure 6C). The results demonstrated that both treatment groups reduced mitochondrial superoxide levels, with the CUR-mEXOs group showing a more pronounced decrease in fluorescence intensity (*p* < 0.05, Figure 6B). Furthermore, spindle integrity is crucial for ensuring normal chromosome segregation during oocyte meiosis (Figure 6G). CUR treatment reduced spindle abnormalities, while CUR-mEXOs further optimized spindle morphology, significantly decreasing the rate of spindle abnormalities (*p* < 0.05, Figure 6H). 

### 3.5. The Effects of CUR and CUR-mEXOs on Mitochondrial Autophagy Factors and Oocyte Maturation Factors

**Figure 7:**
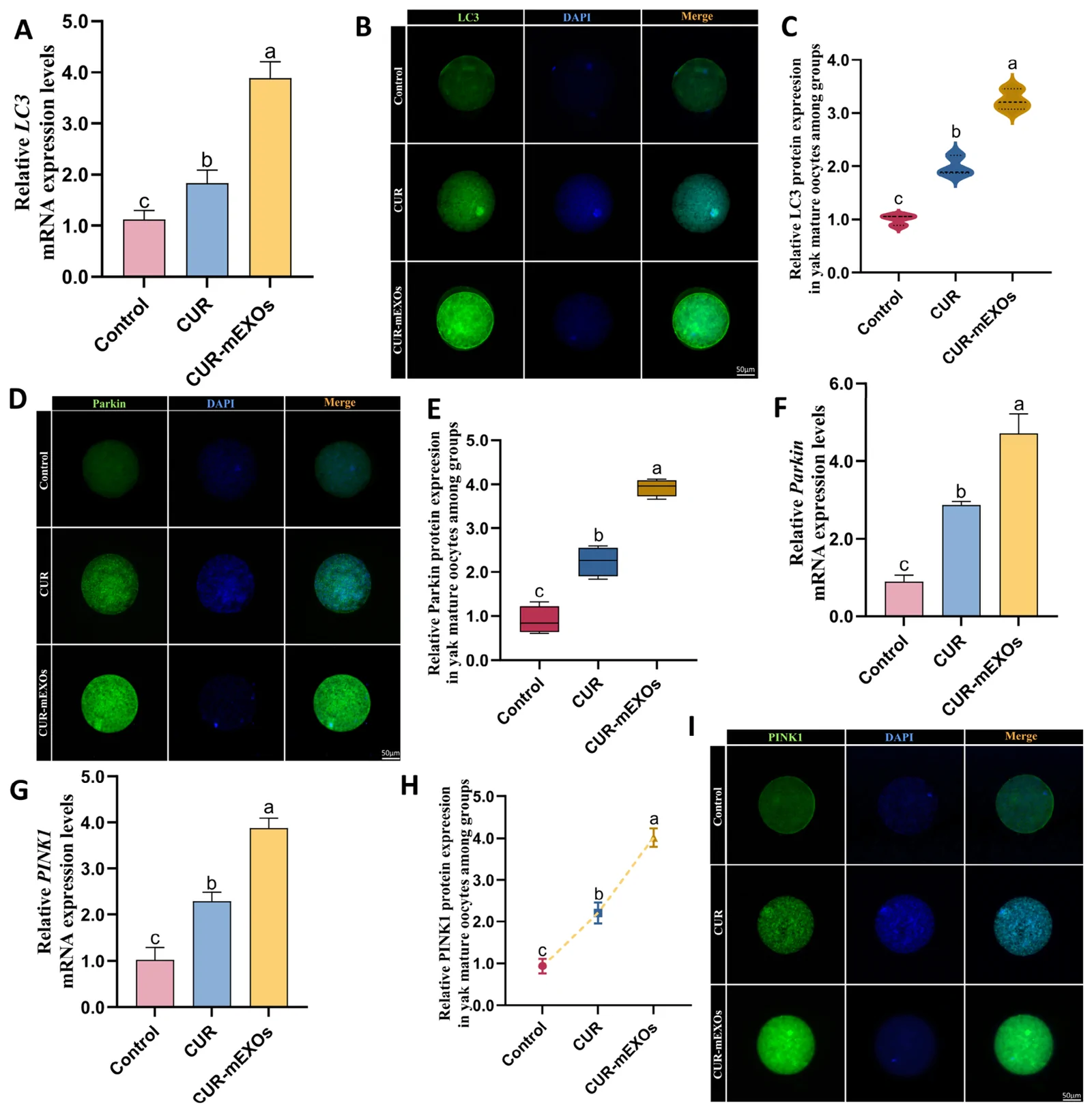
Effects of CUR and CUR-mEXOs on the expression levels of mitochondrial autophagy-related factors. (**A**,**F**,**G**) Relative mRNA expression levels of *LC3*, *Parkin*, and *PINK1* in oocytes from different treatment groups; (**B**,**D**,**I**) Immunofluorescence staining results for LC3, Parkin, and PINK1 protein signals in oocytes of each treatment group; (**C**,**E**,**H**) Quantitative analysis of LC3, Parkin and PINK1 protein abundance in oocytes across all experimental groups. Scale bar = 50 µm. Lines in small trapezoidal plots represent medians. Bars labeled with distinct lowercase letters denote significant statistical discrepancies (*p* < 0.05).

To investigate the effects of CUR and CUR-mEXOs on mitochondrial autophagy levels in yak oocytes, this study examined the expression changes in mitochondrial autophagy-related markers Microtubule-associated protein 1 light chain 3 (LC3), Parkin E3 ubiquitin ligase (Parkin), and PTEN-induced putative kinase 1 (PINK1) (Figure 7). RT-qPCR results indicated that CUR treatment significantly enhanced the expression of *LC3*, *Parkin*, and *PINK1* mRNA, with a more pronounced effect (*p* < 0.05). Additionally, the fluorescence signal intensities of LC3, Parkin, and PINK1 in the CUR-mEXOs group were markedly higher than those in the control and CUR groups (*p* < 0.05), which was consistent with the RT-qPCR findings. We analyzed mitophagy flux at the protein level and performed Peroxisome proliferator-activated receptor gamma coactivator 1-alpha (PGC-1α). staining, revealing markedly elevated PGC-1α fluorescence intensity in the CUR-mEXOs group (Figure S2).

**Figure 8:**
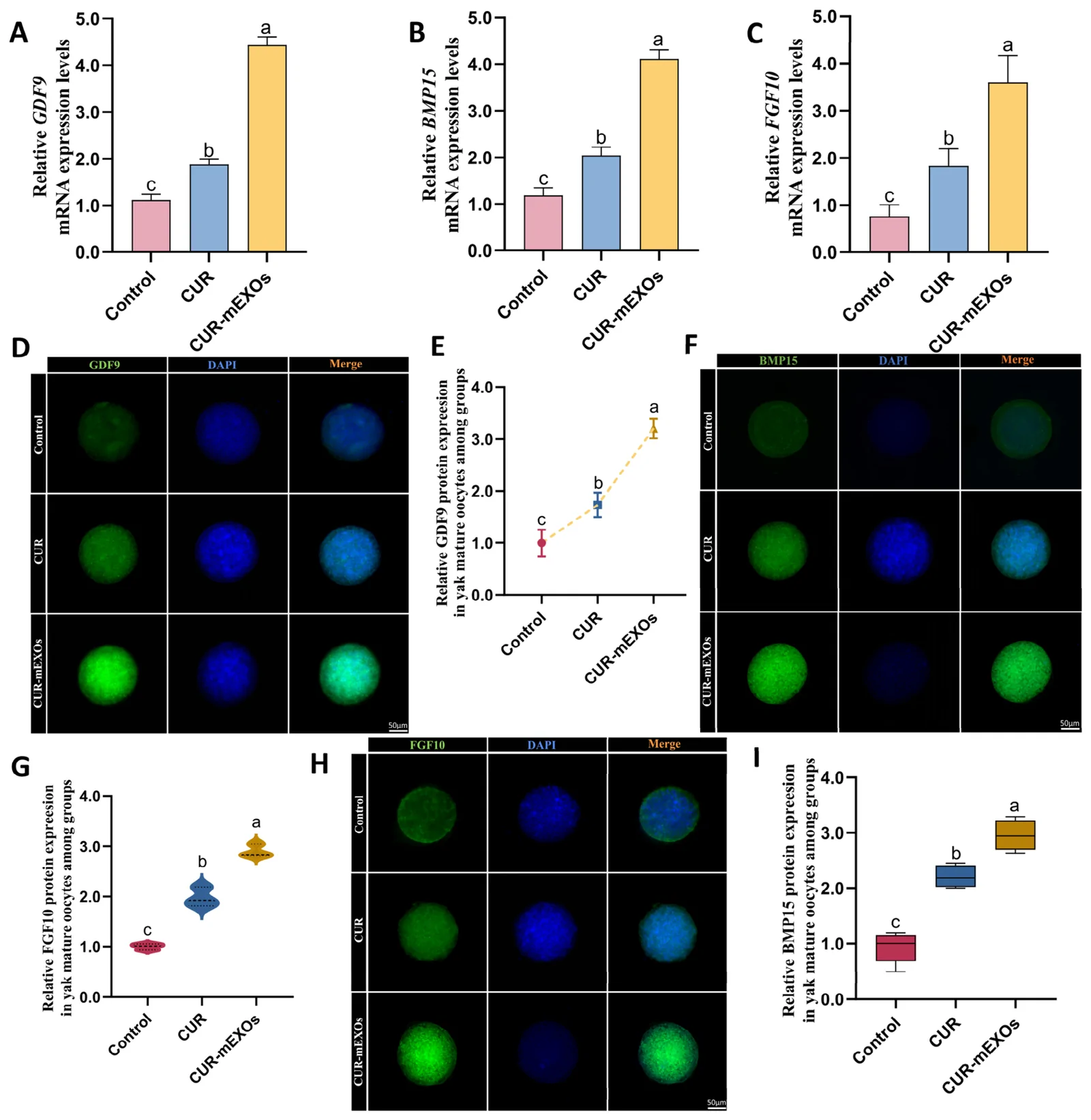
Effects of CUR and CUR-mEXOs on the expression of ovarian-derived factors. (**A**–**C**) Relative mRNA transcript abundance of *GDF9*, *BMP15* and *FGF10* in mature oocytes from various treatment cohorts; (**D**,**F**,**H**) Immunofluorescent staining profiles displaying GDF9, BMP15 and FGF10 protein signals in oocytes under separate interventions; (**E**,**G**,**I**) Quantitative analysis of the relative protein abundance of GDF9, BMP15 and FGF10 among all oocyte treatment groups. Scale bar = 50 µm. Lines in small trapezoidal plots represent medians. Bars labeled with distinct lowercase letters denote significant statistical discrepancies (*p* < 0.05).

Ovarian-derived factors are crucial molecules that regulate the developmental potential of oocytes, the functionality of cumulus cells, and the maturation of the cumulus-oocyte complex. To further evaluate the effects of CUR and CUR-mEXOs on oocyte maturation quality in yaks, this study examined the expression levels of GDF9, BMP15, and FGF10 (Figure 8). RT-qPCR results indicated that CUR treatment significantly upregulated the mRNA expression levels of *GDF9*, *BMP15*, and *FGF10* compared with the control group (*p* < 0.05). Furthermore, the CUR-mEXOs group exhibited a further significant increase in expression levels compared to the free CUR group (*p* < 0.05), suggesting that milk-derived exosomes, as delivery vehicles, can enhance the regulatory effect of CUR on the expression of oogenic factors. Immunofluorescence staining revealed that the fluorescent signals for GDF9, BMP15, and FGF10 were markedly enhanced in the CUR-mEXOs group (*p* < 0.05). 

**Figure 9:**
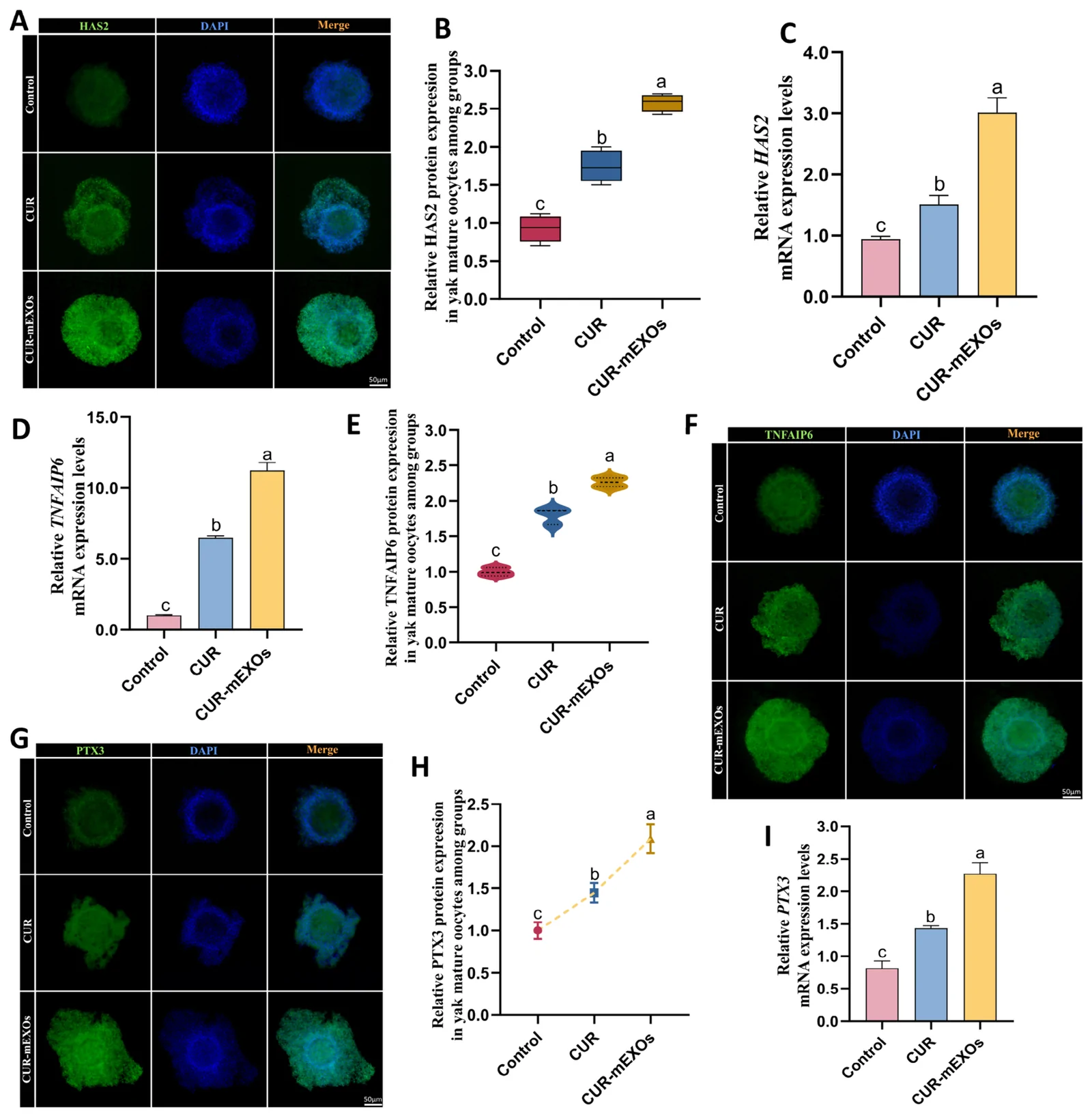
Effects of CUR and CUR-mEXOs on the expression of cumulus expansion factors. (**C**,**D**,**I**) Relative mRNA expression of *HAS2*, *PTX3*, and *TNFAIP6* in oocytes among different treatment groups; (**A**,**F**,**G**) Immunofluorescence staining images showing the protein distribution of HAS2, PTX3, and TNFAIP6 in oocytes from each group; (**B**,**E**,**H**) Quantitative analysis of the relative protein expression levels of HAS2, PTX3, and TNFAIP6 in oocytes across various treatments. Scale bar = 50 µm. Lines in small trapezoidal plots represent medians. Bars labeled with distinct lowercase letters denote significant statistical differences (*p* < 0.05).

HAS2, PTX3, and TNFAIP6 are critical regulatory factors involved in cumulus cell proliferation and extracellular matrix formation. This study further examined the changes in their expression across different treatment groups (Figure 9). The RT-qPCR results indicated that CUR-mEXOs significantly enhanced the mRNA expression of *HAS2*, *PTX3*, and *TNFAIP6*, with the CUR group also demonstrating increased expression compared to the control group (*p* < 0.05). Immunofluorescence analysis of protein expression revealed that, compared to the control group, the fluorescence intensities of HAS2, PTX3, and TNFAIP6 in cumulus cells were elevated in the CUR-treated group. Moreover, the CUR-mEXOs group exhibited stronger fluorescence signals and a larger cumulus cell spreading area (*p* < 0.05). These results were consistent with those of the RT-qPCR analysis. 

In summary, both CUR and CUR-mEXOs were able to improve indicators related to the in vitro maturation of yak oocytes to some extent; however, the CUR-mEXOs group demonstrated more significant regulatory effects on multiple key factors. The RT-qPCR results were consistent with the quantitative immunofluorescence findings, further demonstrating that CUR-mEXOs exhibit stronger biological effects than free CUR.

### 3.6. CUR-mEXOs Regulate Mitochondrial Autophagy and Affect Yak Oocytes

**Figure 10:**
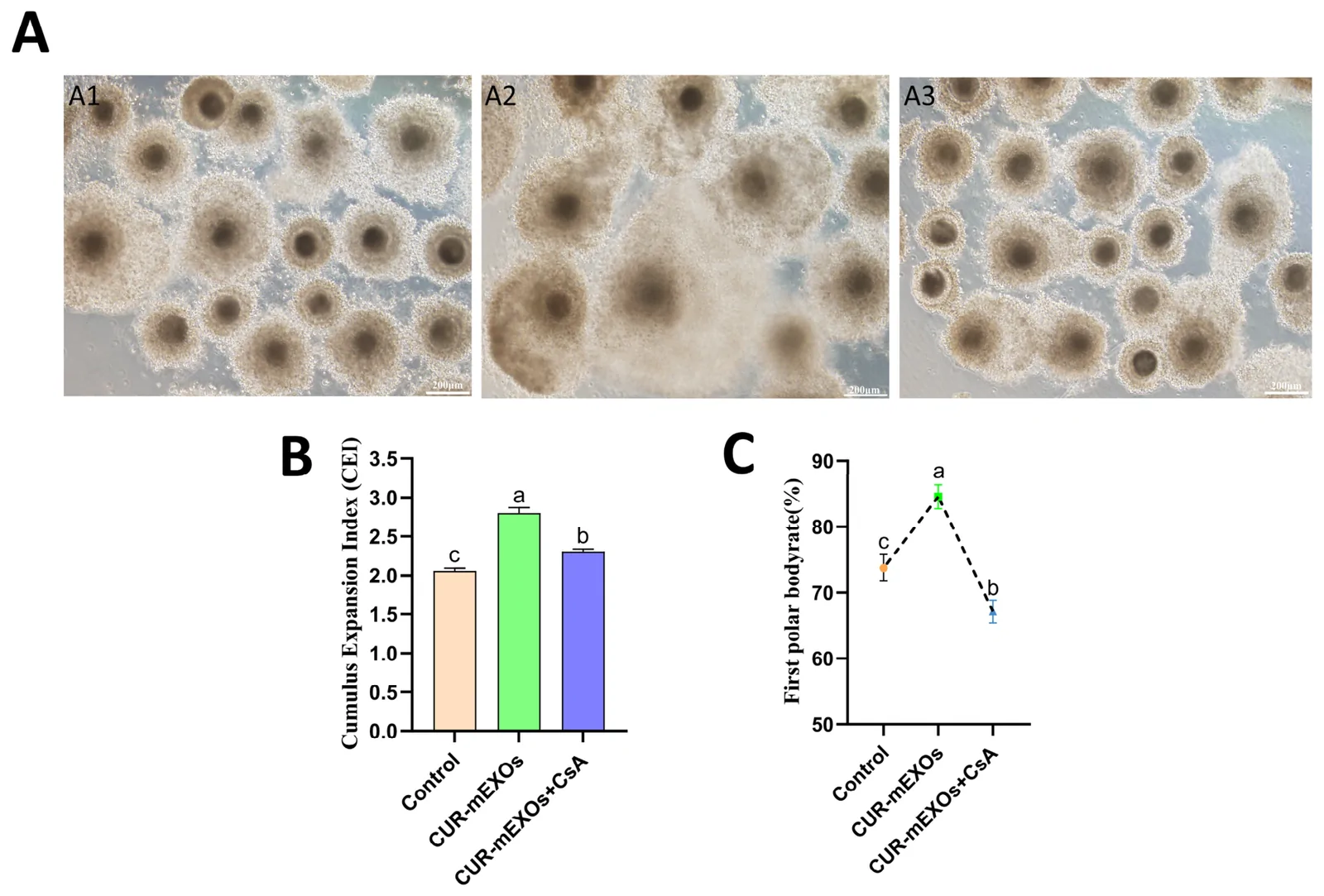
The effect of CUR-mEXOs on mitochondrial autophagy on the cumulus expansion index and first polar body expulsion rate in yak oocytes. (**A**) Typical micrographs exhibiting the morphological characteristics of cumulus expansion in oocytes from distinct experimental groups: (**A1**) Mature COCs in the blank control group; (**A2**) Mature COCs treated with CUR-mEXOs alone; (**A3**) Mature COCs co-treated with CUR-mEXOs and CsA. (**B**) Statistical comparison of cumulus expansion degrees in mature yak oocytes among all groups. (**C**) Number of first polar bodies expelled from oocytes in each treatment group. Scale bar = 200 µm. Bars labeled with distinct lowercase letters denote significant statistical differences (*p* < 0.05).

To investigate whether CUR-mEXOs influence the in vitro maturation of yak oocytes by regulating mitochondrial autophagy, we added a mitochondrial autophagy inhibitor to the culture medium used for the oocytes. Expansion of the cumulus and expulsion of the first polar body were observed 24 h after the initiation of in vitro maturation (Figure 10). It was evident from the data that, compared to the control group, the CUR-mEXOs group exhibited significantly enhanced cumulus expansion and a markedly higher cumulus expansion index. Furthermore, the first polar body expulsion rate in the CUR-mEXOs group was significantly higher than that in the control group (*p* < 0.05, Figure 10B,C). However, upon the addition of the mitochondrial autophagy inhibitor CsA, the oocyte expansion index decreased, and the first polar body expulsion rate declined in the CUR-mEXOs + CsA group compared to the CUR-mEXOs group (*p* < 0.05). This indicates that inhibiting mitochondrial autophagy reduces the beneficial effects of CUR-mEXOs on oocyte expansion and maturation. 

### 3.7. CUR-mEXOs Regulation of LC3, Parkin, PINK1, and Oocyte Maturation Factors via Mitochondrial Autophagy

**Figure 11:**
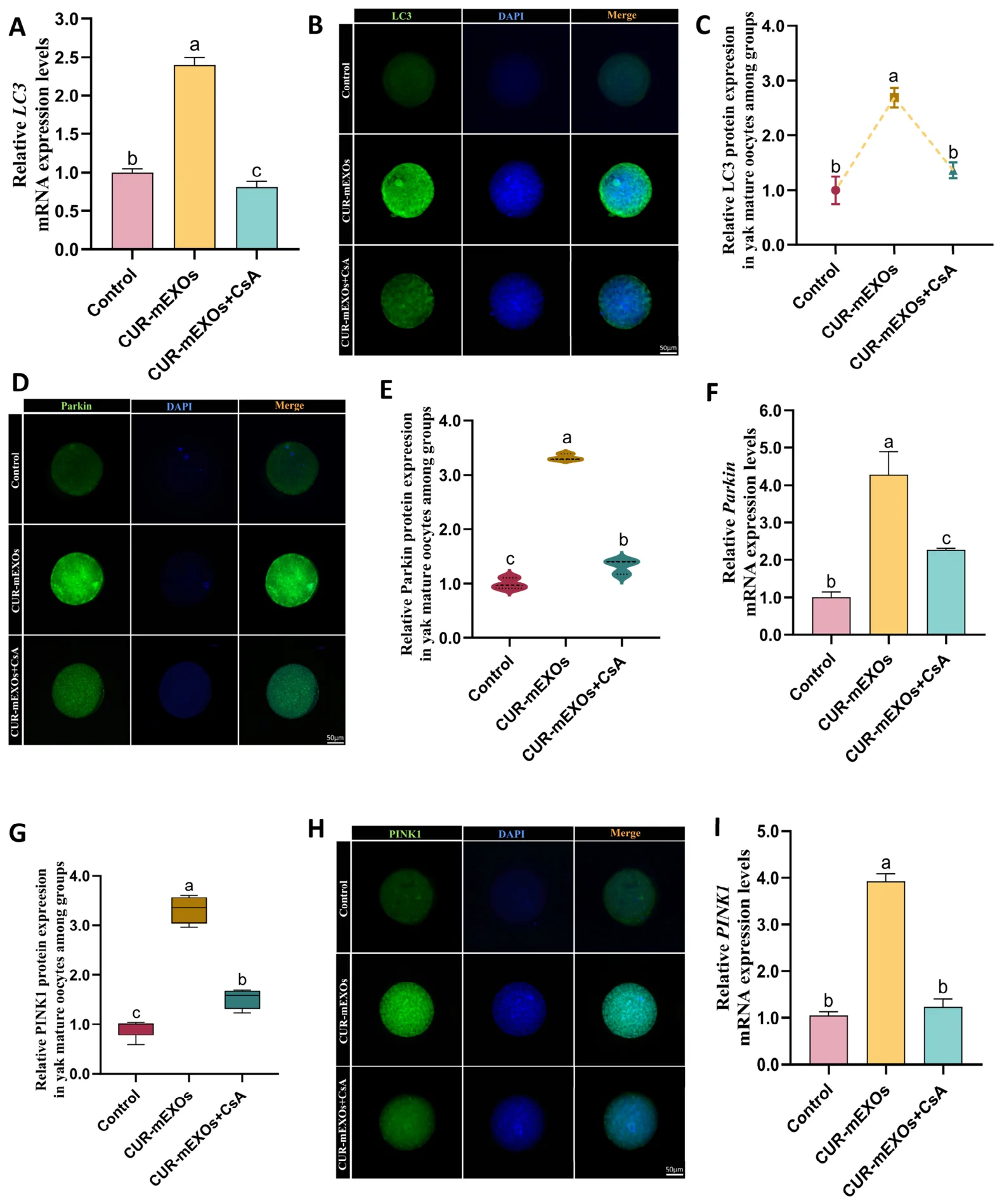
CUR-mEXOs-mediated mitochondrial autophagy regulates the abundance of key mitochondrial autophagy regulators in yak oocytes. (**A**,**F**,**I**) The relative mRNA transcript levels of *LC3*, *Parkin* and *PINK1* were determined in oocytes across different treatment regimens; (**B**,**D**,**H**) Immunofluorescence staining was applied to observe the distribution and expression patterns of LC3, Parkin and PINK1 proteins in oocytes from each experimental group; (**C**,**E**,**G**) Quantitative analysis was performed to evaluate the relative protein expression levels of these three mitochondrial autophagy markers under different culture treatments. Scale bar = 50 µm. Lines in small trapezoidal plots represent medians. Bars labeled with distinct lowercase letters denote significant statistical differences (*p* < 0.05).

To elucidate the regulatory role of CUR-mEXOs in mitochondrial autophagy in yak oocytes, we employed RT-qPCR and immunofluorescence staining to assess the expression levels of mitochondrial autophagy-related markers, namely LC3, Parkin, and PINK1 (Figure 11). The RT-qPCR results indicated that CUR-mEXOs significantly enhanced the transcript levels of mitochondrial autophagy-associated genes; specifically, the mRNA expression levels of *LC3*, *Parkin*, and *PINK1* were markedly increased in the CUR-mEXOs group compared to those in the control group (*p* < 0.05). Following the introduction of the mitochondrial autophagy inhibitor CsA, the mRNA expression levels of these genes in the CUR-mEXOs + CsA group exhibited a significant decrease (*p* < 0.05). Additionally, immunofluorescence staining revealed that the protein fluorescence signal intensities of LC3, Parkin, and PINK1 were significantly higher in the CUR-mEXOs group than in the control group. However, after the addition of CsA, the fluorescence signal intensities in the CUR-mEXOs + CsA group were similarly diminished compared to those in the CUR-mEXOs group (*p* < 0.05). Finally, to further analyze mitochondrial quality control, we performed dual staining with Mito-Tracker and an LC3 antibody (Figure S3). 

**Figure 12:**
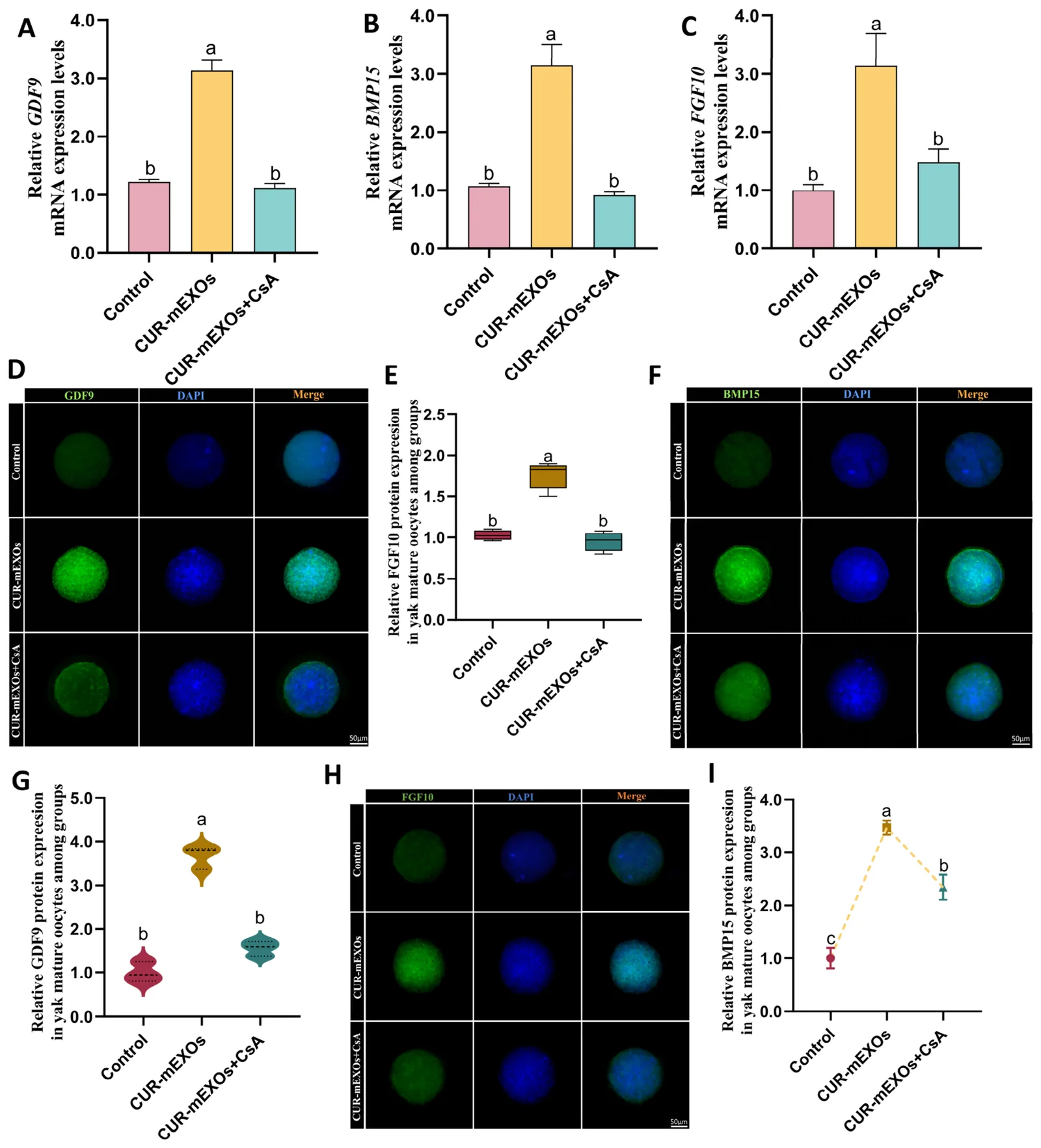
CUR-mEXOs regulate mitochondrial autophagy and influence the expression of ovarian-derived factors in yak oocytes. (**A**–**C**) The mRNA transcript quantities of *GDF9*, *BMP15* as well as *FGF10* were measured in oocyte samples under distinct experimental interventions; (**D**,**F**,**H**) Immunofluorescent labeling images display the localization status of GDF9, BMP15 and FGF10 proteins in oocytes from each experimental cohort; (**E**,**G**,**I**) Quantitative measurements reflect the relative protein contents of GDF9, BMP15 and FGF10 within oocytes of all treatment regimens. Scale bar = 50 µm. Lines in small trapezoidal plots represent medians. Bars labeled with distinct lowercase letters denote significant statistical differences (*p* < 0.05).

To further investigate the effects of CUR-mEXOs on factors related to oocyte development and the regulation of mitochondrial autophagy, we assessed the changes in oocyte-derived factors GDF9, BMP15, and FGF10 (Figure 12). The results indicated that, compared to the control group, the mRNA levels of *GDF9*, *BMP15*, and *FGF10* were significantly increased in the CUR-mEXOs group, with corresponding increases in the intensity of immunofluorescence signals (*p* < 0.05). Following the addition of CsA, both the mRNA levels and fluorescence signal intensities of the aforementioned oocyte-derived factors were markedly downregulated in the CUR-mEXOs + CsA group relative to the CUR-mEXOs group (*p* < 0.05). 

**Figure 13:**
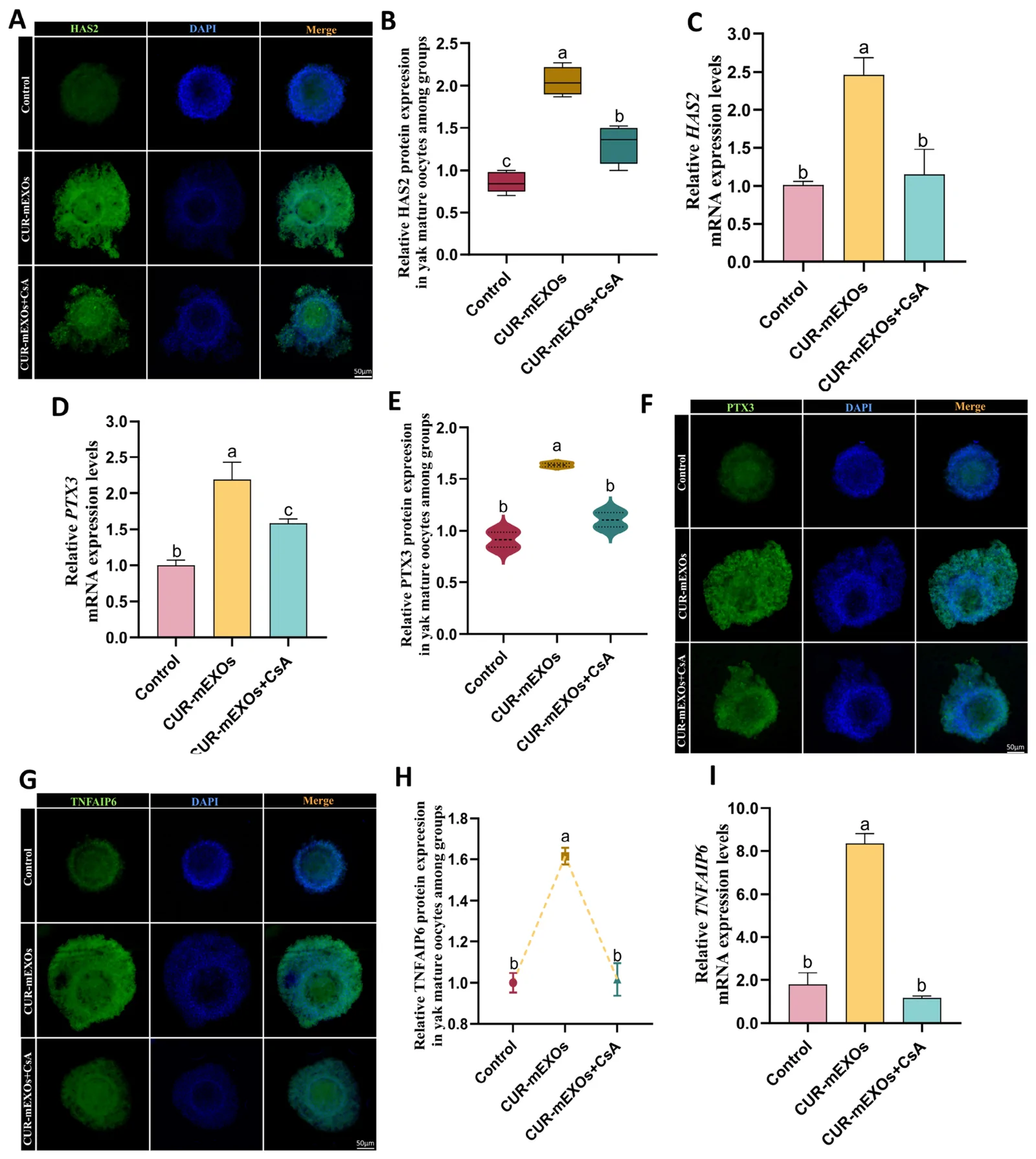
CUR-mEXOs regulate mitochondrial autophagy and influence the expression of factors associated with cumulus expansion in yak oocytes. (**A**,**F**,**G**) Immunofluorescence assay was performed to characterize the endogenous distribution patterns of HAS2, PTX3 and TNFAIP6 proteins within oocyte specimens derived from each experimental cohort; (**B**,**E**,**H**) Quantitative analysis was further conducted to compare the relative protein abundance of the three cumulus expansion-related factors across all treatment regimens; (**C**,**D**,**I**) The mRNA transcript abundances of *HAS2*, *PTX3* and *TNFAIP6* were detected in yak oocytes exposed to diverse culture interventions. Scale bar = 50 µm. Lines in small trapezoidal plots represent medians. Bars labeled with distinct lowercase letters denote significant statistical differences (*p* < 0.05).

Coriolysis is a crucial morphological feature of cumulus-oocyte complex maturation, closely associated with oocyte nutrition, signal transduction, and subsequent fertilization capacity. This study investigated the effects of CUR-mEXOs on factors related to coriolysis following the regulation of mitochondrial autophagy, specifically assessing changes in the expression of HAS2, PTX3, and TNFAIP6 (Figure 13). RT-qPCR results indicated that CUR-mEXOs promoted the expression of genes associated with cumulus expansion. Compared to the blank control cohort, the mRNA expression levels of *HAS2*, *PTX3*, and *TNFAIP6* were evidently upregulated in the CUR-mEXOs group, and the intensity of the immunofluorescence signal was markedly enhanced (*p* < 0.05). In contrast, the CUR-mEXOs + CsA group exhibited reduced mRNA expression levels, with protein expression levels significantly decreased as well (*p* < 0.05). 

### 3.8. CUR-mEXOs Regulate Embryonic Development After Parthenogenetic Activation of Yak Oocytes Through Mitophagy

**Figure 14:**
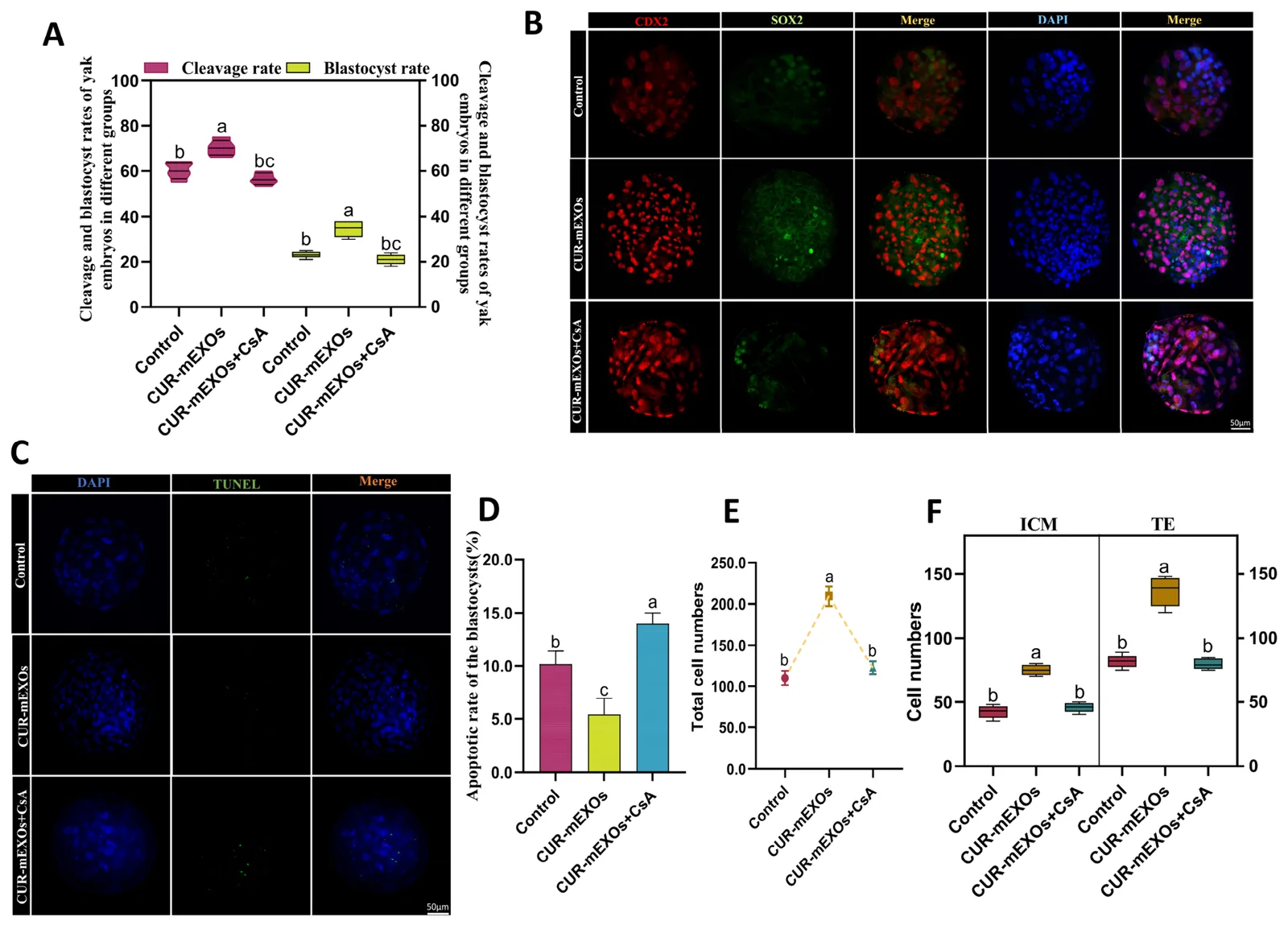
Effects of CUR-mEXOs on cell fate and apoptosis in blastocysts derived from parthenogenetically activated yak oocytes. (**A**) Statistical analysis of cleavage rates and blastocyst development rates in different treatment groups following parthenogenetic activation of oocytes. (**B**) Immunofluorescence labeling was performed to detect CDX2 and SOX2 protein expression in blastocysts generated from parthenogenetically activated oocytes under diverse culture interventions. (**C**) TUNEL fluorescent detection for apoptotic signals within blastocysts originating from parthenogenetically activated oocytes under varied culture regimens. (**D**) Statistical analysis of the apoptosis rate in blastocysts derived from parthenogenetically activated oocytes in different treatment groups. (**E**) Statistical analysis of total cell counts in blastocysts following parthenogenetic activation of oocytes in different treatment groups. (**F**) Number of inner cell mass (ICM) and trophoblast (TE) cells in blastocysts following parthenogenetic activation of oocytes in different treatment groups. Scale bar = 50 µm. Bars labeled with distinct lowercase letters denote significant statistical differences (*p* < 0.05).

This study investigated the potential role of CUR-mEXOs in regulating mitochondrial autophagy during embryonic development following the parthenogenetic activation of yak oocytes. We compared the cleavage and blastocyst formation rates among all experimental cohorts. Compared to untreated cohorts, specimens receiving CUR-mEXOs exhibited significantly elevated cleavage efficiency and blastocyst developmental capacity. In contrast, embryos co-cultured with CUR-mEXOs and CsA displayed distinctly reduced cleavage efficiency and blastocyst developmental capacity relative to the CUR-mEXOs single-treatment cohort (*p* < 0.05, Figure 14A). Subsequent immunofluorescent staining of trophoblast (TE) and inner cell mass (ICM) cells at the blastocyst stage, utilizing CDX2 and SOX2 proteins (Figure 14B), revealed an increase in the total number of blastocyst cells in the CUR-mEXOs group (Figure 14E), with higher counts of both ICM and TE cells. Evidently, oocytes cultured with CUR-mEXOs possessed a considerably higher ICM/TE proportion (*p* < 0.05, Figure 14F). These findings suggest that CUR-mEXOs enhance blastocyst quality and promote ICM fate, thereby increasing the number of ICM cells. The suppression of mitophagy weakened these effects. Moreover, TUNEL staining verified that the apoptotic ratio of blastocysts was distinctly suppressed in the CUR-mEXOs treatment cohort relative to that in the control cohort. In contrast, blastocyst apoptosis levels rose notably in the combined CUR-mEXOs and CsA regimen compared with untreated oocyte specimens (*p* < 0.05, Figure 14C,D). 

## 4. Discussion

Yaks are typical high-altitude livestock species. In vitro maturation of oocytes is susceptible to interference from oxidative stress, metabolic disorders, and microenvironmental imbalances, which significantly affect their developmental quality and embryonic potential [28,29]. Therefore, establishing a safe and efficient optimized in vitro maturation system is crucial for enhancing the efficiency of assisted reproduction in yaks. Although curcumin has been shown to improve oocyte quality, its efficacy is often limited by factors such as stability, bioavailability, and cellular uptake efficiency [30]. In this study, we developed a curcumin nanodelivery system based on milk-derived exosomes (CUR-mEXOs) and applied it for the first time to the in vitro maturation of yak oocytes. The results indicate that owing to their nanoscale structure, natural bilayer lipid membrane properties, and excellent biocompatibility, mEXOs can be effectively taken up by oocytes, significantly improving intracellular delivery efficiency compared to free CUR. This advancement helps overcome the limitations associated with traditional free-drug delivery in in vitro culture systems. Further results indicated that CUR-mEXOs promoted the clearance of damaged mitochondria by regulating mitochondrial autophagy, thereby enhancing the cytoplasmic maturation status of oocytes and consequently improving maturation rates and early embryonic developmental capacity.

This study confirmed that the regulatory effect of CUR on the in vitro maturation of yak oocytes is concentration-dependent, with 10 μM CUR yielding optimal improvement. Low concentrations of CUR may exhibit limited antioxidant effects and fail to effectively alleviate oxidative stress during in vitro culture, whereas high concentrations of CUR may disrupt cellular redox homeostasis and impair oocyte developmental potential. Similar studies on porcine oocytes have confirmed that 10 μM CUR effectively enhances oocyte maturation and embryo development quality, thereby supporting the reliability of the results of the concentration screening in this study [31]. Notably, although CUR has the potential to improve oocyte quality, its low water solubility and bioavailability limit its efficacy [32]. We observed that exosomes were first internalized by outer cumulus cells and then transported into oocytes through gap junctions or transzonal projections. Based on the identification of 10 μM CUR as the optimal concentration, this study established a CUR-mEXOs delivery system. By leveraging the nanoscale structure and lipid bilayer membrane characteristics of exosomes, CUR-mEXOs significantly enhanced the intracellular delivery efficiency of curcumin in oocytes compared to free CUR, thus overcoming the limitations of traditional free drugs in in vitro culture. The experimental results indicated that the oocyte maturation rate, first polar body expulsion rate, and cumulus diffusion index were significantly higher in the CUR-mEXOs-treated group than in the free CUR group. This suggests that bovine-derived exosomes not only enhance drug stability by serving as a physical carrier for CUR but may also improve its biological effects by promoting drug uptake by the cumulus-oocyte complex. Previous studies have reported that bovine-derived exosomes can effectively deliver curcumin, with levels in multiple tissues in vivo being approximately 3–5 times higher than those of free curcumin, which is consistent with the findings of this study [33]. The distinction lies in the fact that previous studies have primarily concentrated on the delivery of substances to tumors [34], inflammatory tissues [35], or mammary tissues [36]. In contrast, this study is the first to introduce a milk-derived exosome delivery system within an in vitro maturation system for yak oocytes, thereby broadening the potential applications of milk-derived exosomes in reproductive biology.

During in vitro maturation, oocytes are extracted from their natural follicular microenvironment, which can lead to the accumulation of reactive oxygen species (ROS) and mitochondrial dysfunction in the oocytes [37,38]. This study demonstrates that CUR-mEXOs significantly reduced intracellular ROS levels in yak oocytes, effectively alleviating oxidative stress damage in vitro, and exhibited markedly superior antioxidant effects compared to free CUR. This suggests that exosome delivery enhances CUR biological activity. Previous research has shown that abnormal intracellular ROS accumulation disrupts mitochondrial function in oocytes, impeding oocyte maturation and subsequent embryonic development, whereas antioxidant treatment can effectively improve oocyte developmental status [39]. These findings align with those of earlier studies, confirming that redox homeostasis is essential for preserving the in vitro developmental potential of yak oocytes. Mitochondria are critical organelles for ATP synthesis, oocyte maturation, and subsequent embryonic development [40]. This study found that CUR-mEXOs can diminish mitochondrial-derived ROS production, indicating that they not only alleviate overall cellular oxidative stress but may also enhance mitochondrial function by reducing mitochondrial oxidative injury. Furthermore, CUR-mEXOs may help maintain mitochondrial homeostasis and improve cytoplasmic maturation quality by upregulating the expression of genes associated with mitochondrial biosynthesis, thereby mitigating excessive ROS accumulation. Consequently, the protective effect of CUR-mEXOs against mitochondrial oxidative damage may represent a fundamental mechanism underlying their capacity to enhance the maturation quality of yak oocytes.

Mitochondrial autophagy plays a crucial role in maintaining mitochondrial function and cellular homeostasis by selectively targeting and degrading damaged mitochondria [41]. Studies have shown that curcumin enhances mitochondrial autophagy in mice via the PINK1/Parkin signaling pathway, mitigating atrazine-induced nephrotoxicity [42]. Additionally, in bovine oocytes, the MitoQ antioxidant has been reported to improve in vitro maturation and embryonic developmental capacity by modulating mitochondrial autophagy [43]. The findings of this study indicate that the mRNA and protein levels of mitochondrial autophagy markers, including *LC3*, *Parkin*, and *PINK1*, were significantly elevated in the CUR-mEXOs treatment group. This suggests that CUR-mEXOs may facilitate oocyte in vitro maturation by promoting the clearance of damaged mitochondria via the regulation of mitophagy. These results imply a close relationship between mitochondrial autophagy-related pathways and the regulation of mitochondrial functions [44]. Furthermore, researchers have discovered that during the vitrification of porcine oocytes, the regulation of PINK1/Parkin-mediated mitochondrial autophagy can restore the embryonic developmental potential of these vitrified oocytes [45]. This study found that moderate activation of mitochondrial autophagy was positively correlated with enhanced expression of oocyte-derived and cumulus diffusion factors. Treatment with CUR mEXOs significantly upregulated the expression of oocyte-derived factors (GDF9, BMP15, and FGF10) and cumulus diffusion factors (HAS2, PTX3, and TNFAIP6), suggesting that mitochondrial autophagy not only regulates intracellular mitochondrial quality but also promotes signal exchange and coordinated maturation between cumulus cells and oocytes [46]. This finding aligns with the results of Zhang et al. in mouse oocytes, which demonstrated that promoting mitochondrial autophagy significantly increased the rate of MII formation and improved the expression of oocyte-derived factors, thereby enhancing oocyte maturation quality [47]. To clarify whether curcumin enhances oocyte maturation by activating mitochondrial autophagy, CsA was used in subsequent experiments. The results indicated that when mitochondrial autophagy was inhibited, the previously observed beneficial effects of CUR-mEXOs were significantly diminished, resulting in reduced oocyte maturation and embryonic developmental capacity. This finding underscores the crucial role of mitochondrial autophagy in promoting oocyte maturation by CUR-mEXOs. These results align with studies conducted on mouse and porcine oocytes, highlighting the significance of dynamic autophagy regulation in the successful completion of meiosis and cytoplasmic maturation [48,49]. In a similar vein, Hou et al. suggested that maintaining a moderate level of activation in the regulation of mitochondrial autophagy is a key mechanism for enhancing oocyte maturation rates, as both excessive and inadequate autophagy can disrupt energy supply and cellular homeostasis [50]. The aforementioned studies further substantiate this viewpoint.

The ultimate indicator of oocyte maturation quality is the subsequent capacity for embryonic development [51]. Some researchers have suggested that the addition of curcumin to bovine sperm cryopreservation and oocyte in vitro maturation systems improves oocyte maturation rates, fertilization rates, and blastocyst formation rates [52]. This study found that after CUR mEXOs activated mitochondrial autophagy, they not only increased cleavage rates and blastocyst formation rates following parthenogenesis but also significantly influenced the differentiation of the inner cell mass and trophoblast cells at the blastocyst stage. Immunofluorescence analysis using specific cellular markers demonstrated that CUR-mEXOs increased the proportion of inner cell mass and maintained the health of trophoblast cells, thereby validating the central role of mitochondrial autophagy in early embryonic development. Similarly, TUNEL results indicated a reduction in apoptosis levels in blastocysts. Previous studies on mouse oocytes have shown that enhancing mitochondrial autophagy and mitochondrial function can improve the developmental capacity of parthenogenetically activated embryos [53]. The present study aligns with these findings and further illustrates that mitochondrial quality control during oocyte maturation continues to influence early embryonic development. Although this study revealed the potential of CUR-mEXOs in oocyte maturation, limitations remain, as in vitro systems cannot fully replicate the complex in vivo environment. Future research should focus on validating the practical efficacy of CUR-mEXOs to provide new insights for optimizing assisted reproductive technologies in high-altitude livestock breeds.

## 5. Conclusions

**Figure 15:**
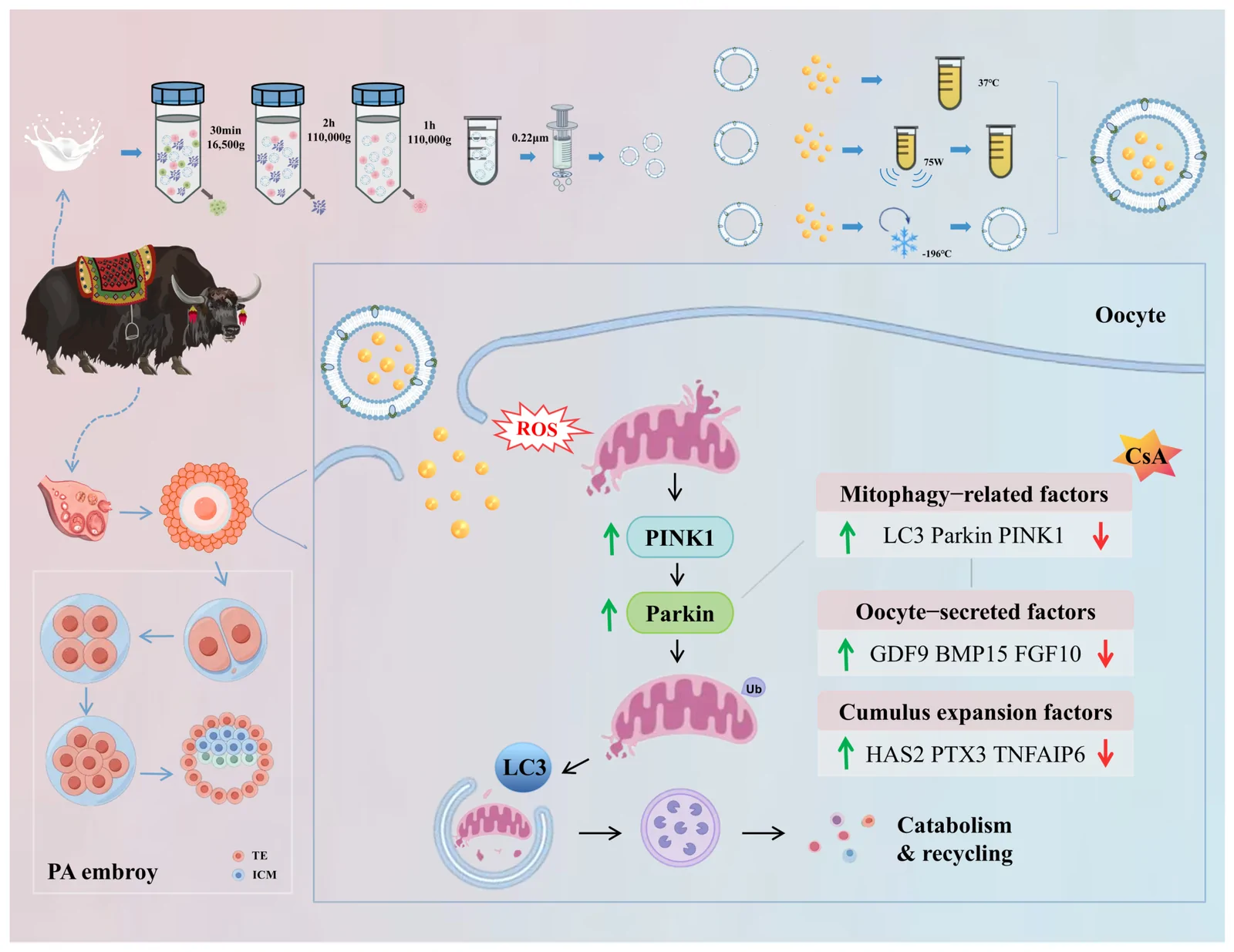
CUR-mEXOs regulate mitochondrial autophagy and influence the expression of oogenic factors in yak oocytes.

In this study, we successfully established a CUR-mEXOs nanodelivery system. The results indicate that CUR-mEXOs significantly improved the quality of oocyte maturation and subsequent developmental potential during in vitro oocyte maturation, achieved through the regulation of mitochondrial autophagy, compared with free CUR (Figure 15). This method optimizes the in vitro maturation system for yak oocytes, enhances embryonic developmental capacity, and provides a reference strategy for improving assisted reproductive technologies for high-altitude and endangered livestock breeds. 

## Figures and Tables

**Table 1:** Primer Information Used in RT-qPCR.

Gene	Primer Sequences 5′–3′	Tm/°C	Accession Number
*GDF9*	F:CAAATGGATTGGATTGATGTG R:GAGCACTTGTGTCGTTCAGATA	58	NM_174681.2
*BMP15*	F: GGCACATACAGACCCTGGACTT R:GAGAGGTGGGAATGAGTTAGGTG	60	NM_001031752.1
*FGF10*	F:GAAGGGGAAACTCTATGGCTCG R: CTATGAGTGTACCACCATCGGAA	58	NM_001206326.1
*HAS2*	F: ACAGGCATCTAACGAACCGAG R: AGTAGGACTTGCTCCAGCGG	60	NM_174079.3
*PTX3*	F: GCTATCGGTCCATAATGCTTG R: CCACCGAGTCACCATTTACC	56	NM_001076259.2
*TNFAIP6*	F:AGCAGTTAGAGGCAGCCAGAAA R:AACACACCACCACACTCCTTTG	61	NM_001007813.2
*LC3*	F: CCGACTTATCCGAGAGCAGC R:TGAGCTGTAAGCGCCTTCTT	59	NM_001001169.1
*Parkin*	F:GTCAATGAGAAGGCGGCAGAGC R:AGGACTTGCGGTTCAGGAGGTT	63	NM_001199065.1
*PINK1*	F:ACATCTCGGCAGGCTCATC R:AGGTGAAGGCTCGCAAGAC	60	NM_001099701.2
*β-* *a* *ctin*	F: GCGGCATTCACGAAACTA R: TGATCTTCATTGTGCTGGGT	56	DQ838049.1

## Data Availability

All data presented in this study are available on reasonable request from the corresponding author. The data are not publicly available due to confidential research restrictions. Interested researchers may contact the corresponding author to obtain all relevant supporting materials.

## Supplementary Materials

The following supporting information can be downloaded at: https://www.mdpi.com/article/10.3390/antiox15080922/s1, Figure S1. Detection of mitochondrial membrane potential in yak oocytes by JC-1 staining; Figure S2. Immunofluorescence staining of PGC-1α in yak oocytes from different treatment groups; Figure S3. Co-localization staining of Mito-Tracker and LC3 for evaluating mitophagy in yak oocytes.
