# Supplementary material for: Curcumin-Loaded Milk-Derived Exosomes Improve the Developmental Competence of Yak Oocytes by Regulating Mitophagy
Source: Antioxidants (Basel). 2026 Jul 24;15(8):922. doi: 10.3390/antiox15080922 (PMC13509650; doi:10.3390/antiox15080922)
Supplement: Supplementary file 1 [file antioxidants-15-00922-s001.zip › antioxidants-4414947-supplementary.pdf]

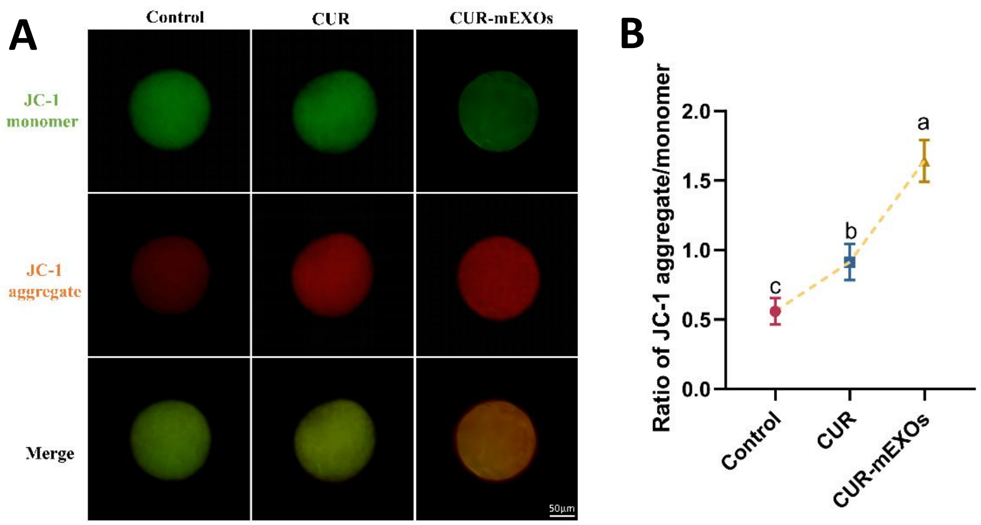

Figure S1. Detection of mitochondrial membrane potential in yak oocytes by JC-1 staining. (A) Representative immunofluorescence images of JC-1 staining in the Control, CUR and CUR-mEXOs groups. (B) Quantitative analysis of the JC-1 aggregate/monomer fluorescence ratio among the three groups. Scale bar = 50 μm. Bars labeled with distinct lowercase letters denote significant statistical differences ($P < 0.05$).

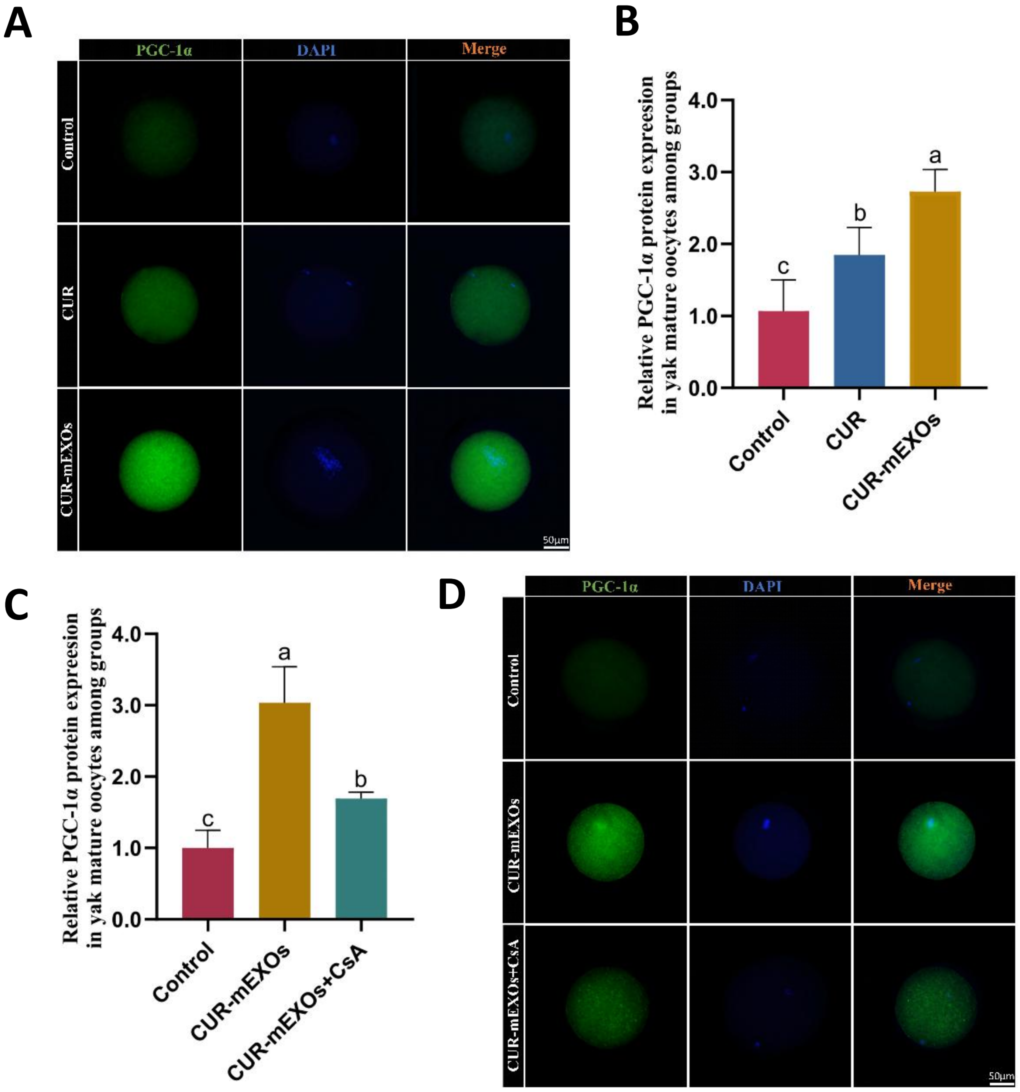

Figure S2. Immunofluorescence staining of PGC-1α in yak oocytes from different treatment groups. (A) Representative immunofluorescence images of PGC-1α in Control, CUR, and CUR-mEXOs groups; (B) Quantitative fluorescence intensity analysis of PGC-1α corresponding to panel (A); (C) Representative immunofluorescence images of PGC-1α in Control, CUR-mEXOs, and CUR-mEXOs + CsA groups. (D) Quantitative fluorescence intensity analysis of PGC-1α corresponding to panel (C). Scale bar = 50 μm. Bars labeled with distinct lowercase letters denote significant statistical differences ($P < 0.05$).

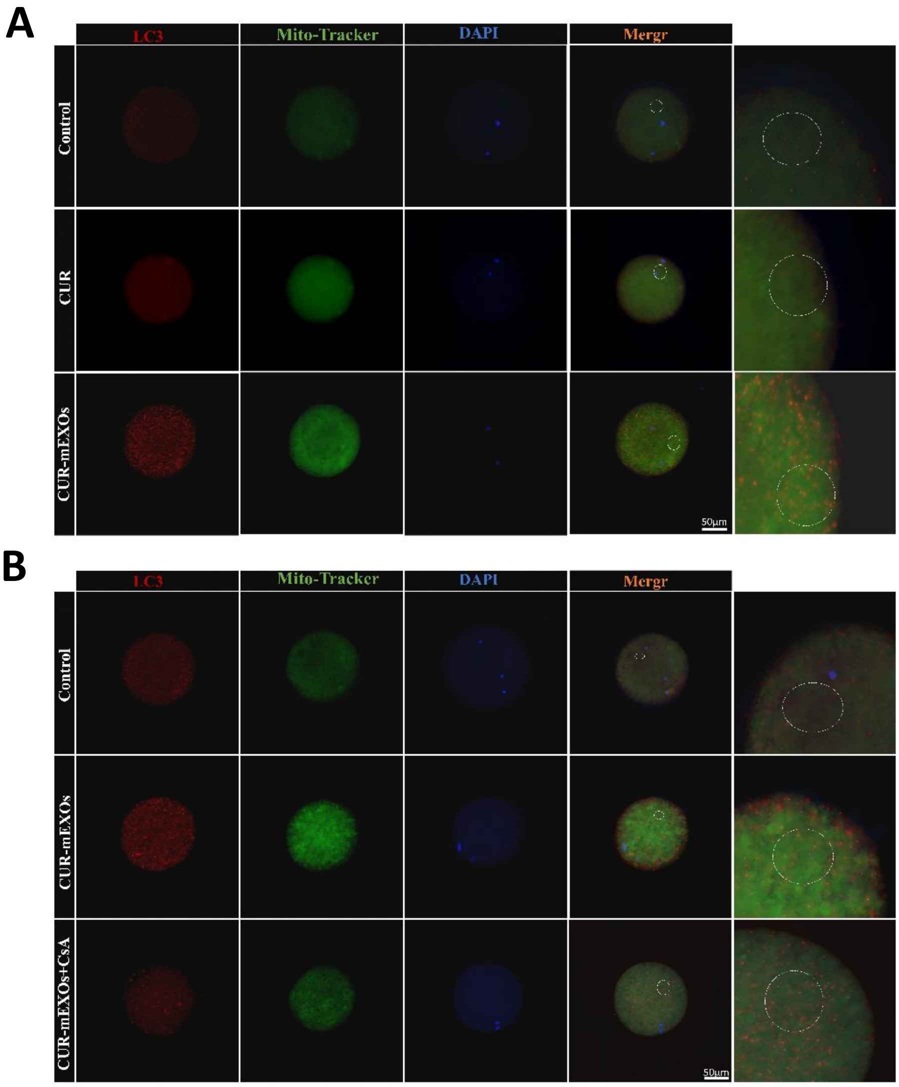

Figure S3. Co-localization staining of Mito-Tracker and LC3 for evaluating mitophagy in yak oocytes. (A) Representative immunofluorescence images of Mito-Tracker and LC3 co-staining in Control, CUR, and CUR-mEXOs groups. (B) Immunofluorescence images of Mito-Tracker and LC3 co-staining in Control, CUR, and CUR-mEXOs groups.
